# The Effect of Three-Monthly Albendazole Treatment on Malarial Parasitemia and Allergy: A Household-Based Cluster-Randomized, Double-Blind, Placebo-Controlled Trial

**DOI:** 10.1371/journal.pone.0057899

**Published:** 2013-03-19

**Authors:** Aprilianto E. Wiria, Firdaus Hamid, Linda J. Wammes, Maria M. M. Kaisar, Linda May, Margaretta A. Prasetyani, Sitti Wahyuni, Yenny Djuardi, Iwan Ariawan, Heri Wibowo, Bertrand Lell, Robert Sauerwein, Gary T. Brice, Inge Sutanto, Lisette van Lieshout, Anton J. M. de Craen, Ronald van Ree, Jaco J. Verweij, Roula Tsonaka, Jeanine J. Houwing-Duistermaat, Adrian J. F. Luty, Erliyani Sartono, Taniawati Supali, Maria Yazdanbakhsh

**Affiliations:** 1 Department of Parasitology, Faculty of Medicine, University of Indonesia, Jakarta, Indonesia; 2 Department of Parasitology, Leiden University Medical Center, Leiden, The Netherlands; 3 Department of Microbiology, Faculty of Medicine, Hasanuddin University, Makassar, Indonesia; 4 Department of Parasitology, Faculty of Medicine, Hasanuddin University, Makassar, Indonesia; 5 Department of Biostatistics, School of Public Health, University of Indonesia, Jakarta, Indonesia; 6 Medical Research Unit, Albert Schweitzer Hospital, Lambaréné, Gabon; 7 Department of Parasitology, Institute of Tropical Medicine, University of Tübingen, Tübingen, Germany; 8 Department of Medical Microbiology, Radboud University Nijmegen Medical Centre, Nijmegen, The Netherlands; 9 Naval Medical Research Unit 2, Jakarta, Indonesia; 10 Department of Gerontology and Geriatrics, Leiden University Medical Center, Leiden, The Netherlands; 11 Department of Experimental Immunology and Department of Otorhinolaryngology, Academic Medical Center, Amsterdam, The Netherlands; 12 Department of Medical Statistics and Bioinformatics, Leiden University Medical Center, Leiden, The Netherlands; Kenya Medical Research Institute - Wellcome Trust Research Programme, Kenya

## Abstract

**Background:**

Helminth infections are proposed to have immunomodulatory activities affecting health outcomes either detrimentally or beneficially. We evaluated the effects of albendazole treatment, every three months for 21 months, on STH, malarial parasitemia and allergy.

**Methods and Findings:**

A household-based cluster-randomized, double-blind, placebo-controlled trial was conducted in an area in Indonesia endemic for STH. Using computer-aided block randomization, 481 households (2022 subjects) and 473 households (1982 subjects) were assigned to receive placebo and albendazole, respectively, every three months. The treatment code was concealed from trial investigators and participants. Malarial parasitemia and malaria-like symptoms were assessed in participants older than four years of age while skin prick test (SPT) to allergens as well as reported symptoms of allergy in children aged 5–15 years. The general impact of treatment on STH prevalence and body mass index (BMI) was evaluated. Primary outcomes were prevalence of malarial parasitemia and SPT to any allergen. Analysis was by intention to treat. At 9 and 21 months post-treatment 80.8% and 80.1% of the study subjects were retained, respectively. The intensive treatment regiment resulted in a reduction in the prevalence of STH by 48% in albendazole and 9% in placebo group. Albendazole treatment led to a transient increase in malarial parasitemia at 6 months post treatment (OR 4.16(1.35–12.80)) and no statistically significant increase in SPT reactivity (OR 1.18(0.74–1.86) at 9 months or 1.37 (0.93–2.01) 21 months). No effect of anthelminthic treatment was found on BMI, reported malaria-like- and allergy symptoms. No adverse effects were reported.

**Conclusions:**

The study indicates that intensive community treatment of 3 monthly albendazole administration for 21 months over two years leads to a reduction in STH. This degree of reduction appears safe without any increased risk of malaria or allergies.

**Trial Registration:**

Controlled-Trials.com ISRCTN83830814

## Introduction

Soil transmitted helminths (STH) (hookworms, *Ascaris lumbricoides* and *Trichuris trichiura*) establish chronic infections in a large proportion of the world population.[1] Major intervention programs using mass drug administration (MDA) to control STH have been launched.[2] However, STH infections seem to persist in the targeted populations raising concern over the development of drug resistance.[3] It is therefore important to conduct well-designed studies that allow evidence-based decisions to be made to maximize effective STH control toward elimination.

While there is no doubt that STH are associated with morbidities in billions of people worldwide, there is also increasing awareness that helminth infections might, like bacterial commensals, play an important role in shaping human health.[4] Helminths may contribute to immunologic and physiologic homeostasis. The immune system is thought to have evolved to operate optimally in the face of helminth-induced immune regulation, and that any disturbance of this long evolutionary co-existence between humans and helminth parasites might be associated with the emergence of pathological conditions[5] possibly involving outcomes of exposure to other pathogens or the development of inflammatory diseases.

In many parts of the world helminths and malarial parasites are co-endemic raising the question of what impact helminth infections may have on the plasmodial parasites that cause malaria. The results have been conflicting in this regard. In some studies a positive association has been reported between helminths and malarial parasitemia while in others, this has been refuted or in yet others a negative association has been shown between helminths and the severity of the clinical outcomes of malaria (reviewed by Nacher).[6]

An increase in the prevalence of allergies has been reported worldwide, in particular in the urban areas of low- to middle-income countries.[7] Although majority of cross-sectional studies have reported inverse associations between helminth infections and allergies[8], [9], two randomized trials with albendazole, have shown conflicting results. One in Ecuador, based on school randomization, reported no change in either SPT reactivity to allergens or allergic symptoms after one year of albendazole treatment[10] while another in Vietnam, in which the randomization unit was individual schoolchildren, showed increased SPT reactivity after one year of albendazole treatment, but consistent with the Ecuadorean study, clinical allergy did not change significantly.[11] It has been suggested that anthelminthic treatment of longer duration might be needed to reveal the modulatory effect of helminths.[12], [13]

In the light of global deworming initiatives, it is important to assess the effectiveness of and to monitor the risks associated with anthelminthic treatment regiments. There is as yet no report of a household-based cluster-randomized double-blind placebo-controlled trial of repeated anthelminthic administration in a community that would be expected to more effectively reduce transmission of STH by decreasing household cross-contamination.

In an area where STH and malaria are co-endemic on Flores Island, Indonesia, we conducted a household cluster-randomized trial of three-monthly albendazole treatment over a two year study period in a whole community to assess benefits and risks associated with this anthelmintic treatment. Specifically we assessed its impact on STH, malarial parasitemia and allergy.

## Methods

### Study population and design

This trial was conducted in two villages in the Ende District of Flores Island, Indonesia (Appendix S1, p2) as described in detail elsewhere.[14], [15] The treatment was based on household and given to all household members except those less than two years old or pregnant (the Indonesian national program guideline). Directly observed treatment was given three monthly during the trial period (June 2008 to July 2010, with treatment starting in Sept 2008). The primary outcomes were prevalence of malarial parasitemia and SPT reactivity to allergens. Additional outcomes were treatment effect on STH and BMI as well as malaria-like and allergy symptoms.

We measured malaria outcomes in Nangapanda only. Malaria was not endemic in Anaranda. Artemisinin-combination therapy (ACT) treatment and treated bed net distribution were not implemented during our study period.[16], [17]

Allergy outcomes were measured, in both villages, in school-age children (5–15 years old) as this group is particularly at risk of developing allergy and asthma[18] and is the target population of global deworming programs.

The study was approved by the Ethical Committee of the Medical Faculty, University of Indonesia (ethical clearance ref: 194/PT02.FK/Etik/2006) and filed by the Committee of Medical Ethics of the Leiden University Medical Center. The trial was registered as clinical trial (Ref: ISRCTN83830814). Prior to the study, written informed consent was obtained from participants or from parents/guardians of children. The study is reported in accordance with the CONSORT guidelines for cluster-randomized studies. The protocol for this trial and supporting CONSORT checklist are available as supporting information; see Checklist S1 and Protocol S1.

### Randomization and masking

The population was randomized by IA using computer aided block randomization at household level utilising Random Allocation software to receive albendazole (single dose of 400 mg) or a matching placebo (both tablets from PT Indofarma Pharmaceutical, Bandung, Indonesia). The treatment code was concealed from trial investigators and participants. The un-blinding of treatment codes occurred after all laboratory results had been entered into the database (August 2011).

### Procedures

Trained community workers measured fever, administered monthly malaria-like symptoms questionnaire which was based on WHO definitions[19] and took finger-prick blood for the three-monthly malarial parasitemia survey. Subjects with fever (≥37.5°C) or additional malaria-like symptoms (headache, fatigue and nausea) at the time of visits were referred to the local primary health centre (puskesmas). Thick and thin Giemsa-stained blood smears were read at University of Indonesia. At baseline, 9 months and 21 months after the first round of treatment blood was collected for PCR-based detection of *Plasmodium spp.* (Appendix S1, p2), a method that is more sensitive than microscopy.[20]

Regarding allergy outcomes, skin prick tests (SPT) with allergens were performed on school-age children in Nangapanda and Anaranda and clinical symptoms of allergy were recorded. House dust mite (*Dermatophagoides pteronyssinus* and *D. farinae*; kindly provided by Paul van Rijn from HAL Allergy Laboratories, Leiden, The Netherlands) and cockroach (*Blatella germanica*; Lofarma, Milan, Italy) were used for SPT which was considered positive with 3 mm cut off.[14] The SPT was performed by one investigator. IgE with specificity for aeroallergens (*D. pteronyssinus* and *B. germanica*) was measured in plasma using an ImmunoCAP 250 system (Phadia, Uppsala, Sweden) following the manufacturer's instructions. All measurements were conducted in one laboratory in the Netherlands. Symptoms of asthma and atopic dermatitis were recorded using a modified visually-assisted version of the International Study of Asthma and Allergy in Childhood (ISAAC) questionnaire as reported before.[14]

Yearly stool samples were collected on a voluntary basis. *Trichuris* was detected by microscopy and a multiplex real-time PCR was used for detection of hookworms (*Ancylostoma duodenale*, *Necator americanus)*, *Ascaris lumbricoides*, and *Strongyloides stercoralis* DNA as detailed before[15] (Appendix S1, p2). Very few subjects were infected with *S. stercoralis* and therefore this infection was not included in analyses.

Body weight and height were measured using the National Heart Lung and Blood Institute practical guidelines (scale and microtoise from SECA GmBH & Co, Hamburg, Germany).

### Power calculation

Sample size estimation was based on the expected change in primary outcomes taking into account a power of 90% and a significance level of <0.05 with a loss to follow-up of 20%. Based on previous observations we expected to find a decrease in malarial parasitemia prevalence and an increase in SPT reactivity after anthelminthic treatment. Based on a prevalence of about 10% and a risk ratio (RR) of 0.60 we aimed to include 2412 people in the malaria assessments. In a pilot study we found SPT to *D. pteronyssinus* to be around 15%, and expected that due to treatment the prevalence would increase. In order to find a RR of 1.5 we aimed to include at least 1418 children.

### Statistical analyses

For children ≤19 years, BMI age-standardized z-scores were calculated according to WHO references.[21] The IgE data were log-transformed to obtain normally distributed variable. To assess treatment effects generalized linear mixed models were used which provide a flexible and powerful tool to derive valid inference while capturing the data correlations induced by clustering within households and repeated evaluations in time of the same subject. Parameter estimates for treatment effects at 9 and 21 months and 95% confidence intervals are reported. The reported p-values are obtained using likelihood ratio tests by comparing the model with and without the treatment effect. Unless stated otherwise all outcomes were adjusted for area (the two study villages in Ende District: Nangapanda or Anaranda) as covariate in the model. For the continuous outcomes (linear mixed-effects models[22] were used with three random effects, namely to model clustering within households a random household specific intercept was used and to model correlation within subjects a random subject specific intercept and slope were used. For the binary outcomes a logistic model was used with random household effects and random subject effects. All models were fitted using the lme4 package (Appendix S1, p6-7).[23] For each fever and additional malaria-like symptoms, total number of events and person months are computed for each treatment arm. Hazard ratios for effect of treatment were calculated with Cox regression with robust SE to allow for within-household clustering (STATA 11).

## Results

**Figure 1:**
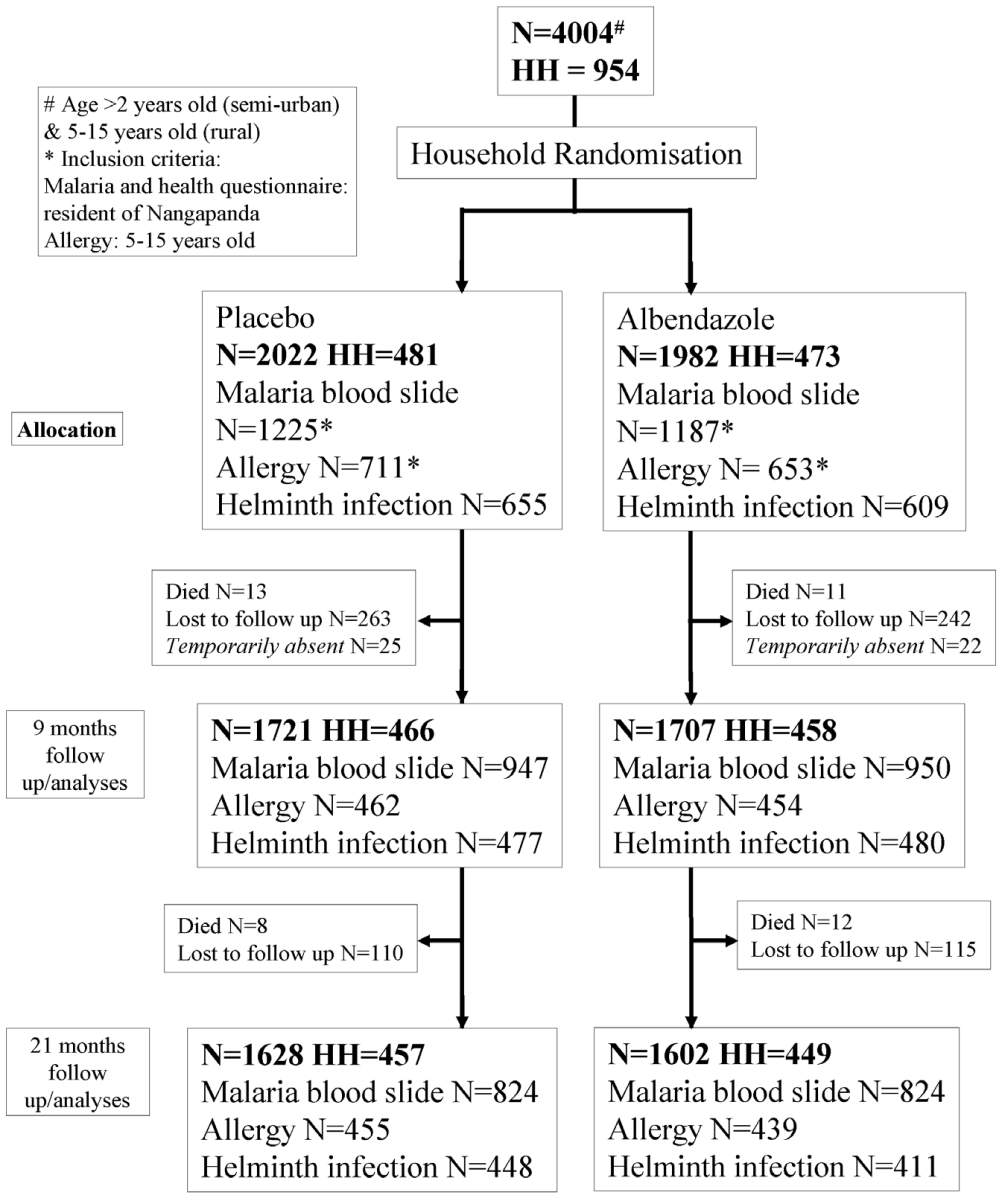
Trial Profile. HH: Household. Lost to follow up implies that the participants have no data from this time point onward. Temporarily absent implies that the participants have no data at this time point but have data available at other time point.

At baseline, 954 households with 4004 subjects were registered. Randomization of households resulted in 1982 people assigned to albendazole treatment and 2022 people to placebo (473 and 481 houses respectively). At baseline 87·3% of the individuals were infected with one or more helminth species. The baseline characteristics were similar between the treatment arms (table 1). The overall trial profile is shown in figure 1, and figure S1A, S1B, S1C (p13–15) in Appendix S1 separately for malaria, allergy and helminth outcomes. The analysis was intention-to-treat and involved all participants as assigned randomly at the start of the trial. During the study, in the albendazole arm 61 people moved to a house that was assigned to placebo while in the placebo arm 62 people moved to a house that was assigned to albendazole. The 44 subjects who died during the trial, included 4 people below the age of 20, 3 between 20 and 40 and the rest above 40 years of age, and were equally distributed between the treatment arms. At 9 months post-treatment full compliance was 77.8% for albendazole treatment and 78.0% for placebo. This was 63.1% and 62.5% respectively at 21 months.

**Table 1:** Baseline characteristics.

		N	Placebo	N	Albendazole
Age (mean in years, SD)		2022	25.7 (18.7)	1982	25.8 (18.7)
Sex (female, n, %)		2022	1090 (53.9)	1982	1042 (52.6)
Area (rural, n, %)		2022	260 (12.9)	1982	253 (12.8)
BMI >19 years old (mean, SD)		575	22.3 (4.0)	582	21.8 (3.6)
Z score of BMI ≤ 19 years old (mean, SD)		427	−1.20 (1.2)	386	−1.37 (1.3)
Parasite infection (n, %)					
Helminth (any *spp*)		655	571 (87.2)	609	533 (87.5)
Hookworm^1^		683	509 (74.5)	629	486 (77.3)
*N. americanus* 1		683	503 (73.7)	629	481 (76.5)
*A. duodenale* 1		683	44 (6.4)	629	41 (6.5)
*A. lumbricoides* 1		683	238 (34.9)	629	209 (33.2)
*S. stercoralis* 1		683	7 (1.0)	629	18 (2.9)
*T. trichiura^2^*		953	258 (27.1)	852	237 (27.8)
Malarial parasitemia (any *spp*)*^2^*		1225	60 (4.9)	1187	52 (4.4)
*P. falciparum*		1225	32 (2.6)	1187	28 (2.4)
*P. vivax*		1225	26 (2.1)	1187	18 (1.5)
*P. malariae*		1225	2 (0.2)	1187	7 (0.6)
Malarial parasitemia (any *spp*)^1^		772	195 (25.3)	739	200 (27.1)
*P. falciparum*		772	106 (13.7)	739	112 (15.2)
*P. vivax*		772	102 (13.2)	739	93 (12.6)
*P. malariae*		772	10 (1.3)	739	18 (2.4)
Skin prick reactivity (n, %)					
Any allergen		711	190 (26.7)	653	163 (25.0)
House dust mite		711	88 (12.4)	653	75 (11.5)
Cockroach		711	163 (22.9)	653	140 (21.4)
Specific IgE, kU/L (median, IQR)					
House dust mite		452	0.8 (0.3–2.6)	431	0.8 (0.2–2.4)
Cockroach		452	1.5 (0.4–5.7)	431	1.9 (0.5–5.0)

1 diagnosed by PCR; ^2^diagnosed by microscopy.

The number of positives (n) of the total population examined (N).

**Figure 2:**
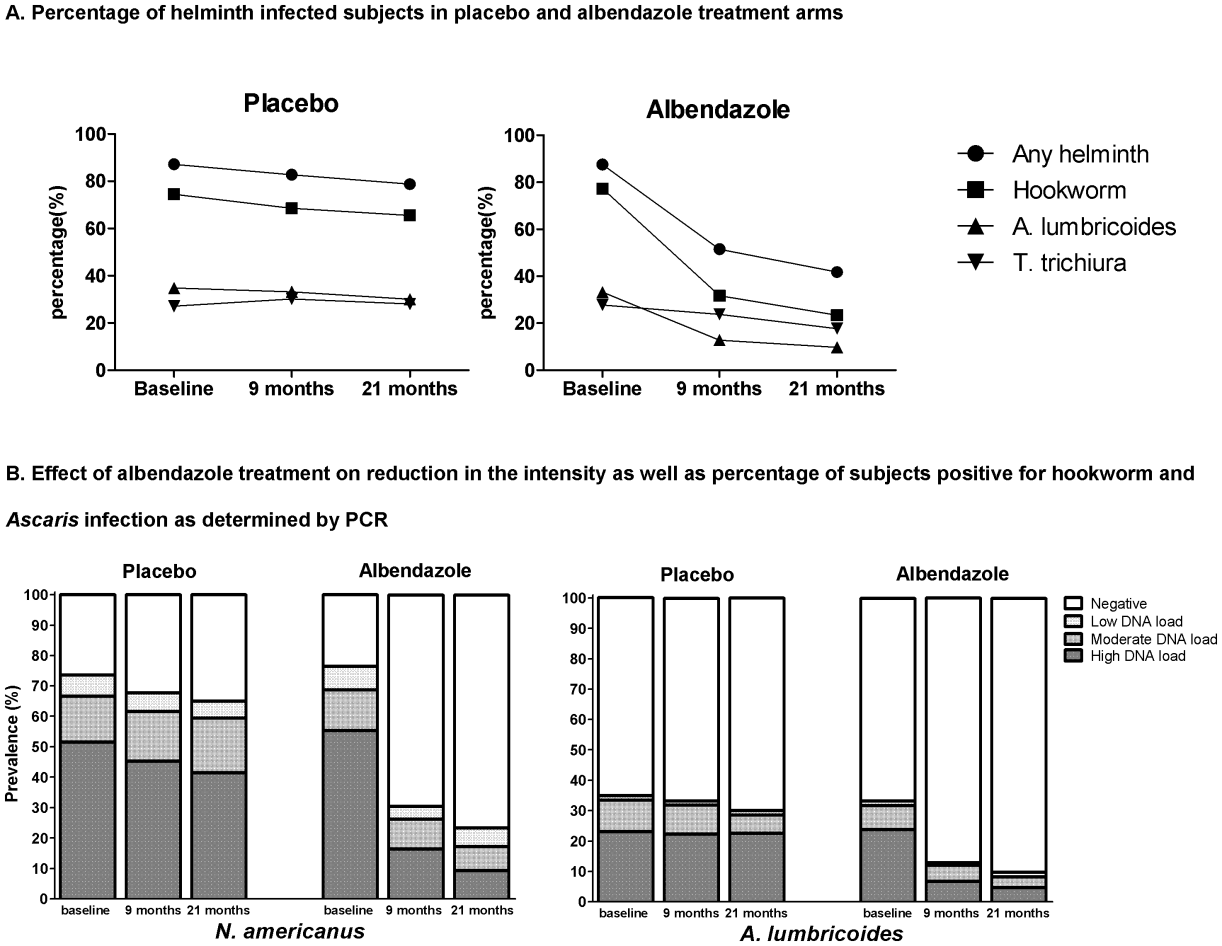
A) Percentage of helminth infected subjects in placebo and albendazole treatment arms. The presence of hookworms (by PCR), *Ascaris lumbricoides* (by PCR) and *Trichuris trichiura* (by microscopy) or any of these helminth infections in subjects who provided stool samples at baseline, 9 and 21 months post treatment (numbers are given in table S1A in Appendix S1). **B**) **Effect of albendazole treatment on reduction in the intensity as well as percentage of subjects positive for hookworm and** ***Ascaris*** **infection as determined by PCR.** Negative is when no helminth specific DNA was found. Positive Ct- values were grouped into three categories: Ct<30.0, 30.0≤Ct<35.0 and ≥35.0 representing a high, moderate and low DNA load, respectively.

This intensive treatment with albendazole resulted in a reduction but not elimination of STH. There was a decrease both after 9 (OR (95% CI) = 0.07 (0.04–0.11) and 21 months (0.05 (0.03–0.08)) of treatment (p<0.0001). Albendazole had the largest effect on hookworm followed by *Ascaris* while the effect on *Trichuris* was less pronounced (figure 2A and table S1 in Appendix S1 p8). Treatment also led to statistically significant reduction in the intensity of hookworm and *Ascaris* infection as determined by PCR (figure 2B). The fact that the stool sampling was on a voluntary basis could have created a selection bias. Analyzing baseline characteristics of subjects providing stool samples and those who did not at 9 months follow up, showed no differences in helminth prevalence, age and sex. Although at 21 months post treatment, sex and helminth prevalence were not different, age was slightly but significantly higher in subjects who provided stool samples mean age in years (SD) = 29.9 (20.4) vs 24.3 (17.5), p = 0.006)

**Figure 3:**
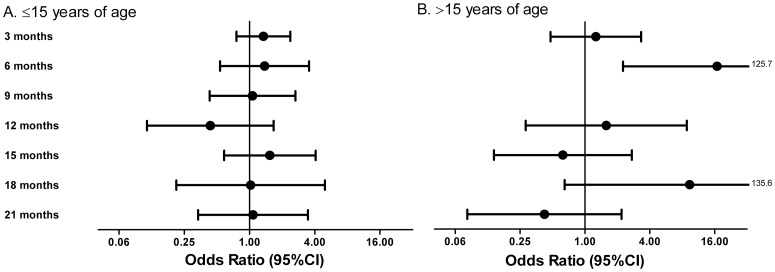
Effect of albendazole treatment on malarial parasitemia based on two age categories. Malarial parasitemia A) ≤15 and B) >15 years of age. The risk of malarial parasitemia after albendazole treatment compared to placebo is shown as odds ratio with 95% CI. The reference line is set at 1, indicating that symbols at the right of this line represent an increased risk, while symbols at the left of the line would predict decreased risk of malarial parasitemia. Note: at 9 month time point in those >15 years of age, the OR is ∞.

The overall percentage of subjects with malarial parasitemia, irrespective of treatment arm, decreased over the trial period (table 2). However, when the data were modelled to assess the effect of albendazole treatment over time, there was a significant (P = 0.0064) increase, which might result from the transient four-fold increased risk of malarial parasitemia (OR 4.16 (1.35–12.80)) (table 3) at 6 months after initiation of treatment (after 2 doses of albendazole). The effect of anthelminthic treatment was assessed in those younger than 15 years of age who would be the prime target of the global deworming programs. The transient increase in parasitemia was only seen in the older (>15 years) age group (figure 3). Malarial parasites were also assessed by PCR, at 9 and 21 months after initiation of treatment and revealed that albendazole had no effect when all *Plasmodium* species were considered together, but when analyzed separately there was a significant increase in the percentage of subjects positive for *P. falciparum* at 9 months post-treatment (table 4). There was no difference in the incidence of fever and additional malaria-like symptoms between the two treatment arms (table S2 in Appendix S1 p10).

**Table 2:** Effect of three-monthly albendazole treatment on malaria outcomes: Percentage of subjects with malarial parasitemia.

	*P. falciparum*		*P. vivax*		*P. malariae*	
	Placebo	Albendazole	Placebo	Albendazole	Placebo	Albendazole
	n/N (%)	n/N (%)	n/N (%)	n/N (%)	n/N (%)	n/N (%)
**Malarial parasitemia by microscopy**						
0 month	32/1225 (2.6)	28/1187 (2.4)	26/1225 (2.1)	18/1187 (1.5)	2/1225 (0.2)	7/1187 (0.6)
3 months	41/897 (4.6)	46/910 (5.1)	17/897 (1.9)	22/910 (2.4)	1/897 (0.1)	6/910 (0.7)
6 months	8/815 (1.0)	20/794 (2.5)	4/815 (0.5)	9/794 (1.1)	0	0
9 months	14/947 (1.5)	7/950 (0.7)	4/947 (0.4)	5/950 (0.5)	1/947 (0.1)	1/950 (0.1)
12 months	9/834 (1.1)	9/813 (1.1)	4/834 (0.5)	2/813 (0.2)	0	0
15 months	14/773 (1.8)	13/772 (1.7)	3/773 (0.4)	4/772 (0.5)	1/773 (0.1)	3/772 (0.4)
18 months	3/815 (0.4)	10/803 (1.2)	1/815 (0.1)	1/803 (0.1)	1/815 (0.1)	1/803 (0.1)
21 months	6/824 (0.7)	11/824 (1.3)	6/824 (0.7)	0	3/824 (0.4)	1/824 (0.1)
**Malarial parasitemia by PCR**						
0 month	106/772 (13.7)	112/739 (15.2)	102/772 (13.2)	93/739 (12.6)	10/772 (1.3)	18/739 (2.4)
9 months	35/656 (5.3)	56/627 (8.9)	56/656 (8.5)	50/627 (8.0)	7/656 (1.1)	9/627 (1.4)
21 months	21/584 (3.6)	31/553 (5.6)	24/584 (4.1)	27/553 (4.9)	10/584 (1.7)	5/553 (0.9)

The number of positives (n) of the total population examined (N).

**Table 3:** Effect of three-monthly albendazole treatment on malaria outcomes: Malarial parasitemia by microscopy

	Placebo	Albendazole	OR (95%CI) ^*^
	n/N (%)	n/N (%)	
Malarial parasitemia (any *spp*)			
3 months	59/897 (6.6)	72/910 (7.9)	1.54 (0.75–3.16)
6 months	12/815 (1.5)	29/794 (3.7)	4.16 (1.35–12.80)
9 months	19/947 (2.0)	13/950 (1.4)	0.57 (0.16–2.04)
12 months	13/834 (1.6)	10/813 (1.2)	0.62 (0.12–3.15)
15 months	18/773 (2.3)	20/772 (2.6)	1.17 (0.18–7.65)
18 months	5/815 (0.6)	12/803 (1.5)	1.84 (0.12–29.03)
21 months	15/824 (1.8)	12/824 (1.5)	0.26 (0.01–6.59)

The number of positives (n) of the total population examined (N). ^*^Odds ratio and 95% confidence interval are based on mixed effects logistic regression models. OR's and 95% CI are shown for the separate time points on malarial parasitemia. The p-value is generated from the modeled data for the combined effect of albendazole treatment over time, which is significant (P = 0.0064) and might result from the effect of 6 months post treatment time point.

**Table 4:** Effect of three-monthly albendazole treatment on malaria outcomes: Malarial parasitemia by PCR.

	Placebo	Albendazole	OR (95% CI)
	n/N (%)	n/N (%)	
Malaria (any spp)			
9 months	95/656 (14.5)	103/627 (16.4)	1.13 (0.77–1.64)
21 months	53/584 (9.1)	59/553 (10.7)	1.09 (0.68–1.76)
*P. falciparum*			
9 months	35/656 (5.3)	56/627 (8.9)	2.82 (1.29–6.15)
21 months	21/584 (3.6)	31/553 (5.6)	1.63 (0.63–4.22)
*P. vivax*			
9 months	56/656 (8.5)	50/627 (8.0)	0.84 (0.41–1.71)
21 months	24/584 (4.1)	27/553 (4.9)	1.40 (0.56–3.52)
*P. malariae*			
9 months	7/656 (1.1)	9/627 (1.4)	0.34 (0.04–2.79)
21 months	10/584 (1.7)	5/553 (0.9)	0.04 (0.00–0.39)

The number of positives (n) of the total population examined (N). Odds ratio and 95% confidence interval based on logistic mixed models. The statistically significant results are given in bold. The p-values are generated from the modeled data for the combined effect of albendazole treatment over time for each of the species separately, which were significant for *P. falciparum* (P = 0.029) and *P. malariae* (P = 0.016).

The proportion of subjects with SPT reactivity was 353/1364 (25.9%) at baseline. Albendazole treatment had no statistically significant effect on SPT to any allergen (table 5), but it was noted that there was an incremental increase in the risk of being SPT positive to any allergen at 9 months and 21 months post initiation of treatment. Moreover, additional analysis on allergens separately, showed a significantly higher SPT to cockroach at 21 months after treatment (1.63 (1.07–2.50)) (table 6). The levels of IgE to allergens showed that albendazole treatment had no effect (table 6). No effect of treatment was seen on symptoms of asthma or atopic dermatitis (table S3 in Appendix S1 p11).

**Table 5:** Effect of three-monthly albendazole treatment on allergy outcomes: Skin prick test to any allergens.

	Placebo	Albendazole	OR (95%CI) ^*^
	n/N (%)	n/N (%)	
SPT to any allergen			
9 months	80/462 (17.3)	82/454 (18.1)	1.18 (0.74–1.86)
21 months	145/455 (31.9)	161/439 (36.7)	1.37 (0.93–2.01)

The number of positives (n) of the total population examined (N). ^*^Odds ratio and 95% confidence interval are based on mixed effects logistic regression models. OR's and 95% CI are shown for the separate time points on SPT to any allergen. The p-value is generated from the modeled data for the effect of albendazole treatment overtime and no significant effects were found (P>0.05).

**Table 6:** Effect of three-monthly albendazole treatment on allergy outcomes: Skin prick test and specific IgE to aeroallergens.

	Placebo	Albendazole	
Skin prick test reactivity^*^	n/N (%)	n/N (%)	OR (95% CI)
House dust mite			
9 months	36/462 (7.8)	35/454 (7.7)	1.31 (0.52–3.27)
21 months	77/455 (16.9)	76/439 (17.3)	1.37 (0.62–3.02)
Cockroach			
9 months	60/462 (13.0)	65/454 (14.3)	1.27 (0.75–2.15)
21 months	112/455 (24.6)	139/439 (31.7)	1.63 (1.07–2.50)
Specific IgE^**^	N (Median, IQR)	N (Median, IQR)	β (95% CI)
House dust mite			
9 months	391 (0.46, 0.16–2.35)	381 (0.46, 0.14–1.98)	1.01 (0.91–1.12)
21 months	339 (0.82, 0.27–3.29)	334 (0.65, 0.20–2.69)	0.93 (0.81–1.06)
Cockroach			
9 months	391 (1.47, 0.30–5.01)	381 (1.55, 0.44–4.40)	1.04 (0.93–1.16)
21 months	339 (1.83, 0.47–5.44)	334 (1.64, 0.42–4.82)	0.98 (0.85–1.14)

The number of positives (n) of the total population examined (N). ^*^Odds ratio and 95% confidence interval based on logistic mixed models; ^**^β (beta) and 95% confidence interval based on generalized linear mixed models from the log-transformed IgE. The values shown are back-transformed. The p-values are generated from the modeled data for the effect of albendazole treatment overtime and no significant effects were found (P>0.05).

No significant change in BMI was observed in children or in adults (table S4 in Appendix S1 p12). Moreover, there was no adverse effect of treatment reported.

## Discussion

This household-based clustered-randomized, double-blind, placebo-controlled trial shows that administering a total of seven single doses of albendazole, at three-monthly intervals, to a population living in an area of Indonesia where STH are highly prevalent, leads to decreased prevalence of helminth infections which although statistically significant, can be taken as an incomplete reduction. The results show a transient increase in malarial parasitemia in the albendazole- compared with the placebo-treated arm in the first six months after initiation of treatment. Albendazole treatment had no statistically significant effect on the designated co-primary outcome, skin prick test reactivity to allergens.

The clinical data collected of fever and additional malaria-like symptoms, were not affected by the deworming. Clinical signs of asthma and atopic dermatitis were also not affected by albendazole treatment.

The prevalence of infection was high (>60%), which reflects the situation in many areas that are being targeted by the global deworming programs. Using a three-monthly treatment regimen which represents an extreme scenario for helminth control strategy, percentage of subjects positive for STH was reduced by 39% compared to placebo. It should be noted that in our study the sensitive PCR method has been used. The reduction in the proportion of subjects infected with hookworm and *Ascaris* was more pronounced than for *Trichuris* infections, confirming the findings using a single dose of albendazole.[24] Subjects who provided stool samples at 21 months were slightly but significantly older than those who did not. Given that hookworm infection is more prevalent in older subjects, this may have contributed to the poor deworming achieved by albendazole. The reduction achieved in worm loads, did not have any beneficial effect on BMI. Observational studies have reported that helminth infections affect growth; however randomized trials have not been consistent.[25], [26] In this regard, our study would support the outcome of a recent Cochrane review of no beneficial effect of deworming programs on nutritional indicators [27] even though it can be argued that in our study the suboptimal reduction in the STH would not allow any beneficial effect of anthelmintic in terms of BMI to be seen in the community. Importantly, the fact that the effect of such an intensive deworming strategy in a community is incomplete, needs to be considered in the agenda for the control and elimination of helminth diseases of humans.[28]

Most studies on the effect of helminth infections on malarial parasitemia and clinical malaria episodes have used cross-sectional designs and have been inconclusive.[6] Longitudinal studies of anthelminthic treatment have also reported conflicting results.[29], [30] A small study conducted in Madagascar[29] has reported an increase in malarial parasitemia in levimasole treated subjects, older than 5 years of age, while in Nigeria [30], albendazole treatment of pre school children was associated with lower *P. falciparum* infection and anemia, however, the lost to follow up in this study was very high. The question whether albendazole treatment during pregnancy could affect health outcomes in the offspring, was addressed in a recent report from Uganda.[31] It was found that the incidence of malaria up to one years of age was not different in the offspring of mothers born to those treated with albendazole or placebo. Our study, reports the results of a community wide randomized-controlled trial that used three-monthly malarial parasitemia data obtained by microscopy. A significantly higher percentage of subjects positive for malarial parasites in the albendazole compared to the placebo arm was seen but this seemed to be a transient effect and restricted to individuals older than 15 years of age, an age group that is not the main target of the current deworming programs. The question arises as to why this effect was only seen in those >15 years of age. This could be due to the fact that *Ascaris* infection is lower in older age and therefore more easily cleared. It has been suggested that *Ascaris* is the spesies of helminth that has the most effect on malarial parasitemia and diseases.[6] Therefore by removing *Ascaris* in older age, we might be seeing a more profound effect on malarial parasitemia.

Using PCR, which enables detection of sub-microscopic infections at species level, it was also concluded that albendazole did not affect overall malarial parasitemia. When malaria species were analyzed separately, the percentage of subjects infected with *P. falciparum* but not with *P. vivax* increased significantly in the first 9 months post-treatment in the albendazole-treated arm, which is contrary to our hypothesis that anthelminthic treatment would reduce prevalence of malarial parasitemia.[32] It was expected that by decreasing STH, the immune hyporesponsiveness would be reversed and this would be associated with stronger immune effector responses to malaria parasites. One of the possible explanations for the enhanced malarial parasitemia would be that with a reduction in STH, there is increased nutrient availability for other co infections and their growth.

It has been suggested that there are different malaria outcomes with different species of helminths; *Ascaris* being associated with protection regarding parasitemia and severity of malaria while hookworm with higher incidence of malaria.[6] Our study was not powered to conduct a stratified analysis, and with the overall gradual decrease in malaria in the study area during our study, the numbers of subjects positive for malaria parasites are too few for an ad hoc analysis.

The findings concerning allergy outcomes, although not significant, are in line with our hypothesis that anthelminthic treatment would increase SPT reactivity. The risk of SPT reactivity increased incrementally with longer treatment and raises the question whether even longer deworming periods are needed for more pronounced effects on allergic outcomes. In support of this, a recent study reported that 15–17 years of ivermectin treatment for onchocerciasis control in Ecuador led to a significant increase in SPT reactivity to allergens.[12] In the same country, one year of anthelmintic treatment in schoolchildren did not lead to any change in SPT.[10] The question whether different species of helminths might affect allergic outcomes to a different degree, remains unanswered. It is interesting that a one year anthelmintic treatment in Vietnam where hookworm infection was the prominent species, as in our study, resulted in a significant increase in SPT positivity in schoolchildren. This is in contrast to what was seen in Ecuador where *Ascaris lumbricoides* was the most prevalent species. One common feature of the anthelmintic trials seems to be that clinical symptoms of allergy do not change with deworming. However, an important trial in pregnant women in Uganda has shown an increased risk of infantile eczema in infants of mothers treated with anthelminthics compared to those that received placebo.[33] This could indicate that exposure to worms in early life, might affect allergic outcomes more profoundly than when helminths are removed later in life.[34]

One of the limitations of this trial is the overall decrease in malarial parasitemia during the two year study period, most probably caused by actively referring subjects with malaria-like symptoms to puskesmas. Therefore further studies in areas highly endemic for malaria are needed. Treatment in the trial did result in a significant reduction in percentage of subjects infected with STH, but this reduction was incomplete. It is therefore possible that the community was insufficiently dewormed. However, our primary aim was to measure the possible effect of deworming programmes on malaria or allergy. We conclude that despite transient increase in malarial parasitemia as a result of albendazole treatment, there were no clinically relevant changes to outcome measures 21 months after treatment was initiated.

In conclusion, an extremely intensive anthelminthic treatment in a community where STH are highly endemic, does not lead to elimination but reduces both prevalence and intensity of helminths. Such MDA regiment appears safe and does not lead to any significant change with respect to malaria infections or allergies. However, it is worrying that such vigorous community treatment does not have a more pronounced effect on STH burden. Better integrated control strategies would be needed to deworm and subsequently assess whether the risk for malaria infections or allergies change.

## Supporting Information

Appendix S1 **Here we describe additional details on methods: study area and procedure, data collection on clinical symptoms, and detailed description of statistical models used.** We provide tables on the effect of three monthly albendazole treatment on helminth infections, on reported fever and malaria-like symptoms, on reported clinical symptoms of allergy, and on body mass index. In addition, trial profiles of the separate outcomes are provided: malarial parasitemia, skin prick test, and helminth infection.
(DOC)

Checklist S1 **CONSORT Checklist.**
(DOC)

Protocol S1 **Trial Protocol.**
(DOC)
