# Correction: The Effect of Three-Monthly Albendazole Treatment on Malarial Parasitemia and Allergy: A Household-Based Cluster-Randomized, Double-Blind, Placebo-Controlled Trial

**DOI:** 10.1371/annotation/fd262a2e-9dc2-40de-ac18-a46d5b92ce9e

**Published:** 2013-09-09

**Authors:** Aprilianto E. Wiria, Firdaus Hamid, Linda J. Wammes, Maria M. M. Kaisar, Linda May, Margaretta A. Prasetyani, Sitti Wahyuni, Yenny Djuardi, Iwan Ariawan, Heri Wibowo, Bertrand Lell, Robert Sauerwein, Gary T. Brice, Inge Sutanto, Lisette van Lieshout, Anton J. M. de Craen, Ronald van Ree, Jaco J. Verweij, Roula Tsonaka, Jeanine J. Houwing-Duistermaat, Adrian J. F. Luty, Erliyani Sartono, Taniawati Supali, Maria Yazdanbakhsh

A contribution of the sixth author (MAP) to the study was incorrectly omitted from the Contributions Statement. The following should be included with the Author Contributions Statement: "Contributed to the follow up data collection: MAP." 

