# Supplementary material for: The Effect of Three-Monthly Albendazole Treatment on Malarial Parasitemia and Allergy: A Household-Based Cluster-Randomized, Double-Blind, Placebo-Controlled Trial
Source: PLoS One. 2013 Mar 19;8(3):e57899. doi: 10.1371/journal.pone.0057899 (PMC3602425; doi:10.1371/journal.pone.0057899)

**Supplementary Appendix S1**

**Table of contents**  p 1

1. Supplementary methods p 2

Additional information on the study area and procedures p 2

Data collection on clinical symptoms p 4

Detailed description of the statistical models used p 6

2. Supplementary tables p 8

Table S1. Effect of three-monthly albendazole treatment on helminth infection p 8

Table S2. The effect of albendazole on fever and additional malaria-like

symptoms p 10

Table S3. Reported clinical symptoms of allergy p 11

Table S4. Effect of three-monthly albendazole treatment on BMI p 12

3. Supplementary figures p 13

Figure S1A. Profile of trial with malarial parasitemia as outcome in the village of

Nangapanda where malaria is endemic p 13

Figure S1B. Profile of trial with skin prick test (SPT) reactivity as outcome in

children 5-15 years of age in both Nangapanda and Anaranda p 14

Figure S1C. Profile of trial with helminth infection as outcome in villages of

Nangapanda and Anaranda p 15

**Supplementary Methods**

Additional information on the study area and procedures

Ende district, an area highly endemic for STH, is situated near the equator (8^o^45’S, 121^o^40’E) and it is characterized by a uniform high temperature, in the range of 23-33.5 °C, with humidity of 86-95%. Average yearly rain fall is 1.822 mm with about 82 rainy days, especially from November to April, with the peak in December until March. The semi-urban village of Nangapanda, endemic for malaria, had a population of 3583 and is located in the coastal area with most villagers being farmers and fishermen with some government officers or private sector employees. The rural village Anaranda had 1631 inhabitants and is located 80 km further inland of Nangapanda. There was poor infrastructure and inhabitants generated income mainly from farming.

Regarding the availability of the anthelminthics in the community, there was no deworming campaign in this area during the study period. Pyrantel pamoat (Combantrin®) and dehydropiperazine (Bintang 7 puyer 17®) were the only available anthelminthics in the market. The local primary health centre (Puskesmas) did not provide the current trial study participants by any anthelminthic treatment but referred them to the trial team.

Malaria control, such as by artemisinin-combination therapy (ACT) treatment and insecticide-treated nets (ITN) or long-lasting insecticide-treated nets (LLIN) although planned, were not implemented yet during our study period. This was due to several difficulties faced in some parts of Indonesia, such as instable drug supply, lack of training on definitive diagnosis of malaria by the laboratory staff, as well as insufficient bednet supply and poor compliance.^17^ Malaria drugs such as chloroquine and quinine were available in the shops, however, little information is available on proper self medication. Therefore, before and during the study period, regular training of field workers was undertaken on how to prevent malaria (use of repellent and bednet, irrigation of breeding places) and how to treat malaria (not to self medicate but to visit puskesmas for diagnosis and treatment). Indoor residual spraying was done by the local health authority for dengue control against an outbreak at the beginning of 2008.

The treatment of suspected malaria cases at the puskesmas was chloroquine and primaquine for *P. vivax,* while for *P. falciparum* sulfadoxine/pyrimethamine was commonly used. Subjects in our study with fever and/or any one of the malaria-like symptoms (see below for detailed description) were referred to the puskesmas for assessment and treatment according to local health center policy.

The anthelmintic treatment and placebo were coded and the code was concealed from trial investigators and participants. The tablets were distributed by trained health workers and the intake was directly observed. Labels with the study subject ID were printed from a computer database and attached to the appropriate strip of treatment by a separate team located in Jakarta without the involvement of the study investigators. In order to assess whether anthelminthic treatment had any adverse effect on the growth of children or on the incidence of allergy, interim analyses were done at one year post-treatment by a monitoring committee. After completion of the study the whole population was treated with albendazole (a single dose of 400mg for three consecutive days).

The malaria slides were read by microscopy at the Department of Parasitology in Jakarta. The quality control for microscopic reading took place in the pilot phase of the project. In cooperation with NAMRU-2 (US Naval Medical Research Unit-2) two microscopists from our team were trained, inter-observer differences were assessed and following satisfactory training they were certified. At the pilot phase, and throughout the study, PCR was used to monitor the microscopy data with a high degree of agreement between microscopy and PCR. In a random sub-sample at 9 months and 21 months post-treatment we measured malarial parasitemia by PCR.

Primers and the *P. falciparum*, *P. vivax*, *P. ovale*, and *P. malariae*-specific probes were used with some modifications in the fluorophore- and quencher-chemistry. Amplification reactions of each DNA sample are performed in white PCR plates, in a volume of 25 μl with PCR buffer (HotstarTaq master mix), 5 mmol/l MgCl2, 12.5 pmol of each Plasmodium-specific primer and 15 pmol of each PhHV-1-specific primer, 1.5 pmol of each *P. falciparum*, *P. vivax*, *P. malariae*-specific XS- probes, and PhHV-1-specific Cy5 double-labelled detection probe, and 2.5 pmol of each *P. ovale*-specific XS-probes (Biolegio), and 5 μl of the DNA sample were used. Amplification consists of 15 min at 95ºC followed by 50 cycles of 15 s at 95ºC, 30 s at 60ºC, and 30 s at 72ºC. Amplification, detection, and analysis are performed with the CFX96 real-time detection system (Bio-Rad laboratories). The PCR output from this system consists of a cycle-threshold (Ct) value, representing the amplification cycle in which the level of fluorescent signal exceeds the background fluorescence, and reflecting the parasite-specific DNA load in the sample tested. Negative and positive control samples are included in each amplification run.

Stool samples were collected and preserved in 4% formaldehyde for microscopy examination or frozen (-20°C) unpreserved for PCR detection. The formol-ether acetate concentration method was performed on the formalin preserved stool samples followed by microscopic examination for *Trichuris trichiura* infections. As described in detail before,^15^ DNA was isolated from approximately 100 mg unpreserved feces and examined for the presence of *Ancylostoma duodenale, Necator americanus, Ascaris lumbricoides* and *Strongyloides stercoralis* DNA by the multiplex qPCR. The qPCR output from this system consisted of a Ct value; negative and positive control samples were included in each run of the amplification. Positive Ct- values were grouped into three categories: Ct<30.0, 30.0≤Ct<35.0 and ≥35.0 representing a high, moderate and low DNA load, respectively.

Data collection on clinical symptoms

A year before the study enrolment, community workers were recruited and trained in taking finger-prick blood for the three-monthly malarial parasitemia survey in Nangapanda, observing drug intake, recording adverse treatment effects, as well as measuring fever and administering monthly malaria-like symptoms questionnaire. These questionnaires were based on WHO definitions^19^ and were assessed in all individuals that were present at the time of the survey. Subjects with fever (≥37.5^o^C) or additional malaria-like symptoms (headache, fatigue and nausea) at the time of visits were referred to the puskesmas for treatment according to local standard protocols. The monthly data on fever (≥37.5, using digital thermometer) and additional malaria-like symptoms were collected at baseline September 2008 and in the months Oct 08, Nov 08, Dec 08, Jan 09, Feb 90, March 09, Apr 09, May 09, June 09, Aug 09, Sept 09, Oct 09, Nov 09, Dec 09, Jan 10, Feb 10, March 10 and Apr 10. At baseline, 1396 individuals were assessed in placebo and 1381 in the albendazole arm and at the last timepoint, 1165 and 1181 subjects were followed up in the two groups, respectively. Questionnaire data were available for all timepoints from 45.8% and 47.2% of placebo and albendazole group whereas data for 80% of the timepoints were available from 83.8% and 87.6% of the two groups, respectively. The number of events was recorded in total of 15259 and 15307 person months at risk for placebo and albendazole groups, respectively.

The modified video-assisted (for asthma symptoms) and illustration-assisted (for atopic dermatitis) ISAAC questionnaire, translated to Bahasa Indonesia and back translated for use in our studies within the EU funded project GLOFAL ([www.glofal.org](http://www.glofal.org/)), were administered at baseline and at 21 month timepoints. Data were available from 629 in placebo and 635 in albendazole arm at baseline, while these numbers were 460 and 445, respectively, at the 21 month timepoint. These questionnaires were administered to the parents/guardians of subjects who were skin prick tested with allergens: the trial profile is given in supplementary figure 1B. The prevalence of asthma symptoms were obtained from the following questions: (i) has your child ever had asthma? (ii) has your child ever been diagnosed for asthma by a doctor? and (iii) has your child in the past 12 months had wheezing or whistling in the chest?; while the prevalence of atopic dermatitis was obtained from the questions: (i) has your child ever had doctor/paramedic diagnosed allergic eczema and (ii) has your child ever had one or more skin problems accompanied by an itchy rash?

If the answer to one or more of these questions was positive, the subjects were considered to have either asthma or atopic dermatitis symptoms.

Detailed description of the statistical models used

Descriptives were computed for each variable (mean and standard deviation or median and interquartile range for continuous outcomes, numbers and percentages for categorical variables). For children ≤ 19 years, BMI age-standardized z-scores were calculated according to WHO references.^21^

Two sources of correlation among observations should be accounted for when modeling these data, namely observations at various timepoints for a subject are correlated due to subject specific effects and observations within households are correlated due to environmental effects shared within households. To model these correlations we used random effects. For subject effects a random intercept and a random slope were used, i.e. each subject has its own intercept and slope, where the latter models the change of the outcome variable over time. Observations within a household also have a shared random intercept. Thus the intercept for an observation of a specific subject from a specific household is the overall mean plus the subject specific effect plus the household effect. By doing so correlation among observations of the same household was modeled since these observations share the same household effect. To assess treatment effects generalized linear mixed models^22^ were used where the term “mixed” corresponds to the used random effects. Unless stated otherwise all models included area as covariate in the model to take into account the differences between the two villages. Generalized linear mixed models provide a flexible and powerful tool to derive valid inference while capturing the data correlations induced by clustering within households and repeated evaluations in time of the same subject.

For continuous outcome variables which were measured at 0, 9 and 21 months, treatment effects were modeled at timepoint 9 and 21 months, because treatment started at 0 months and the design is a randomized trial no treatment effect should be present at time 0. We allowed for different treatment effects at 9 and 21 months. Beta’s and 95% confidence intervals are provided for 9 and 21 months. The betas represent the mean difference between the placebo and treatment group. An overall test for treatment effect over time was performed by using a likelihood ratio test which compares the model with and without the treatment effect (2 df test).

For binary outcome variables measured at 9 and 21 months, the logit link was used (mixed effect logistic regression). In these models only the two random intercepts were included and the random subject specific slope was omitted. Odds ratios and 95% confidence intervals are reported. Analogously to continuous outcome variables two degrees of freedom likelihood ratio tests were performed to assess treatment effects over time. Note that the model based odds ratios are different from crude odds ratios directly computed from the sample due to missing observations and due to the presence of random effects and the covariate area in the model. Malarial parasitemia by microscopy was measured at a three monthly basis. To model these data, similar models were used. Specifically at each of the seven timepoints (excluding time zero) a treatment effect was included. The likelihood ratio test for treatment effect over time has therefore 7 degrees of freedom. All generalized linear mixed models were fitted using the lme4 package (Douglas Bates, Martin Maechler and Ben (2011). lme4: Linear mixed-effects models using S4 classes. R package version 0.999375-42. http://CRAN.R-project.org/package=lme4) in R.^23^

For each malaria-like symptom (fever, headache, fatigue, and nausea), total number of events and person months were computed for each treatment group. We calculated incidence rates for all events. Symptom episodes within three months of an initial presentation with the same symptom were regarded as part of the same episode. Hazard ratios for effect of treatment were calculated with Cox regression with robust SE to allow for within-subject and within household clustering (STATA 12).

**Supplementary Tables**

**Table S1. Effect of three-monthly albendazole treatment on helminth infection**

|  | **Placebo** | **Albendazole** | **OR (95% CI)** |
| --- | --- | --- | --- |
|  | n/N (%) | n/N (%) |
| Helminth infection (any spp) |  |  |  |
| 9 months | 395/477 (82.8) | 247/480 (51.4) | 0·07 (0·04-0·11) |
| 21 months | 353/448 (78.8) | 172/411 (41.9) | 0·05 (0·03-0·08) |
| Hookworm^1^ |  |  |  |
| 9 months | 359/524 (68.5) | 161/508 (31.7) | 0·02 (0·01-0·04) |
| 21 months | 305/466 (65.5) | 99/423 (23.4) | 0·01 (0·01-0·03) |
| *A. lumbricoides^1^* |  |  |  |
| 9 months | 174/524 (33.2) | 65/508 (12.8) | 0·24 (0·16-0·36) |
| 21 months | 140/466 (30.0) | 41/423 (9.7) | 0·18 (0·11-0·29) |
| *T. trichiura^2^* |  |  |  |
| 9 months | 219/726 (30.2) | 160/673 (23.8) | 0·58 (0·42-0·80) |
| 21 months | 177/633 (28.0) | 101/571 (17.7) | 0·40 (0·28-0·58) |

The number of positives (n) of the total population examined (N).^1^diagnosed by PCR. ^2^diagnosed by microscopy. Odds ratio and 95% confidence interval based on logistic mixed models. The p-values are generated from the modeled data for the combined effect of albendazole treatment over time, which were significant (P < 0.001) for any helminth and for each of the species separately.

**Table S2. The effect of albendazole on fever and additional malaria like symptoms**

|  | **Placebo** | | **Albendazole** | | **Unadjusted IRR** | **Adjusted IRR** |
| --- | --- | --- | --- | --- | --- | --- |
|  | **Events**  (PM) | **Incidence**  per PM | **Events**  (PM) | **Incidence**  per PM |
| Fever | 414 (18494) | 0.02 | 429 (18636) | 0.02 | 1.03 | 1.03 |
| Headache | 333 (19067) | 0.02 | 340 (19563) | 0.02 | 1.00 | 1.00 |
| Fatigue | 49 (22362) | 0.002 | 69 (22535) | 0.003 | 1.39 | 1.41 |
| Nausea | 76 (21749) | 0.003 | 55 (22211) | 0.002 | 0.71 | 0.71 |
| Any symptom | 661 (15259) | 0.04 | 690 (15307) | 0.05 | 1.04 | 1.04 |

IRR: incidence rate ratio

PM: Person months

Adjusted with age and sex

The p-values are generated from Cox regression of albendazole treatment over time with robust SEs to allow for within-subject and within household clustering and no significant effects were found (P>0.05).

**Table S3**. Reported clinical symptoms of allergy

|  | **Placebo** | **Albendazole** | **OR (95% CI)** |
| --- | --- | --- | --- |
|  | n/N (%) | n/N (%) |
| **Asthma** |  |  |  |
| 21 months | 8/461 (1.7) | 11/445 (2.5) | 1.11 (0.07-17.26) |
| **Atopic dermatitis** |  |  |  |
| 21 months | 13/461 (2.8) | 9/445 (2.0) | 0.57 (0.16-2.02) |

The number of positives (n) of the total population examined (N).The p-values are generated from the modeled data for the effect of albendazole treatment after 21 months and no significant effects were found (P>0.05).

At baseline 8/692 (1.2%) and 18/692 (2.6%) in the placebo group reported symptoms of asthma and atopic dermatitis, respectively, while in Albendazole this was 10/635 (1.6%) and 11/635 (1.7%).

**Table S4. Effect of three-monthly albendazole treatment on BMI**

|  | **Placebo** | **Albendazole** | **β (95% CI)** |
| --- | --- | --- | --- |
|  | N (Median, IQR) | N (Median, IQR) |
| **BMI** |  |  |  |
| 9 months | 498 (22.42, 19.91 - 25.54) | 499 (22.07, 19.96 - 24.56) | -0·10 (-0·29-0·09) |
| 21 months | 430 (22.42, 19.68 - 25.56) | 425 (21.56, 19.44 - 24.12) | -0·15 (-0·39-0·10) |
| **z-BMI** |  |  |  |
| 9 months | 346 (-0.81, -1.44 - -0.13) | 334 (-0.96, -1.56 - -0.30) | -0·04 (-0·17-0·09) |
| 21 months | 272 (-1.29, -2.21 - -0.56) | 269 (-1.57, -2.32 - -0.74) | -0·07 (-0·23-0·10) |

The total population examined (N). IQR = Interquartile range. β (beta) and 95% confidence interval based on generalized linear mixed models. The p-values are generated from the modeled data for the combined effect of albendazole treatment over time and no significant effects were found (P>0.05). Baseline data are shown in Table 1 of the manuscript.

**Supplementary Figures**

Figure S1A. Profile of trial with malarial parasitemia as outcome in the village of Nangapanda where malaria is endemic


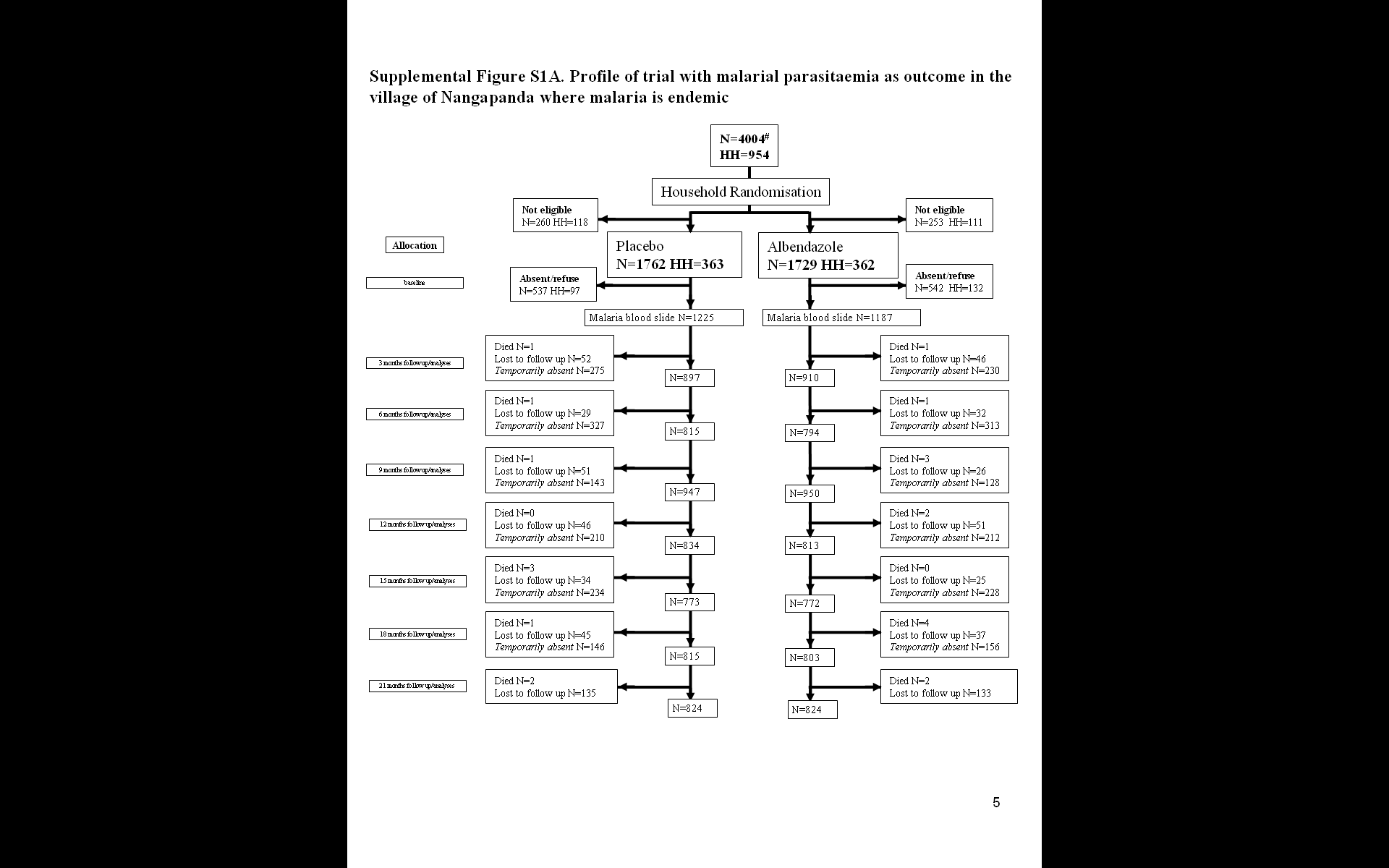


Figure S1B. Profile of trial with skin prick test (SPT) reactivity as outcome in children 5-15 years of age in both Nangapanda and Anaranda


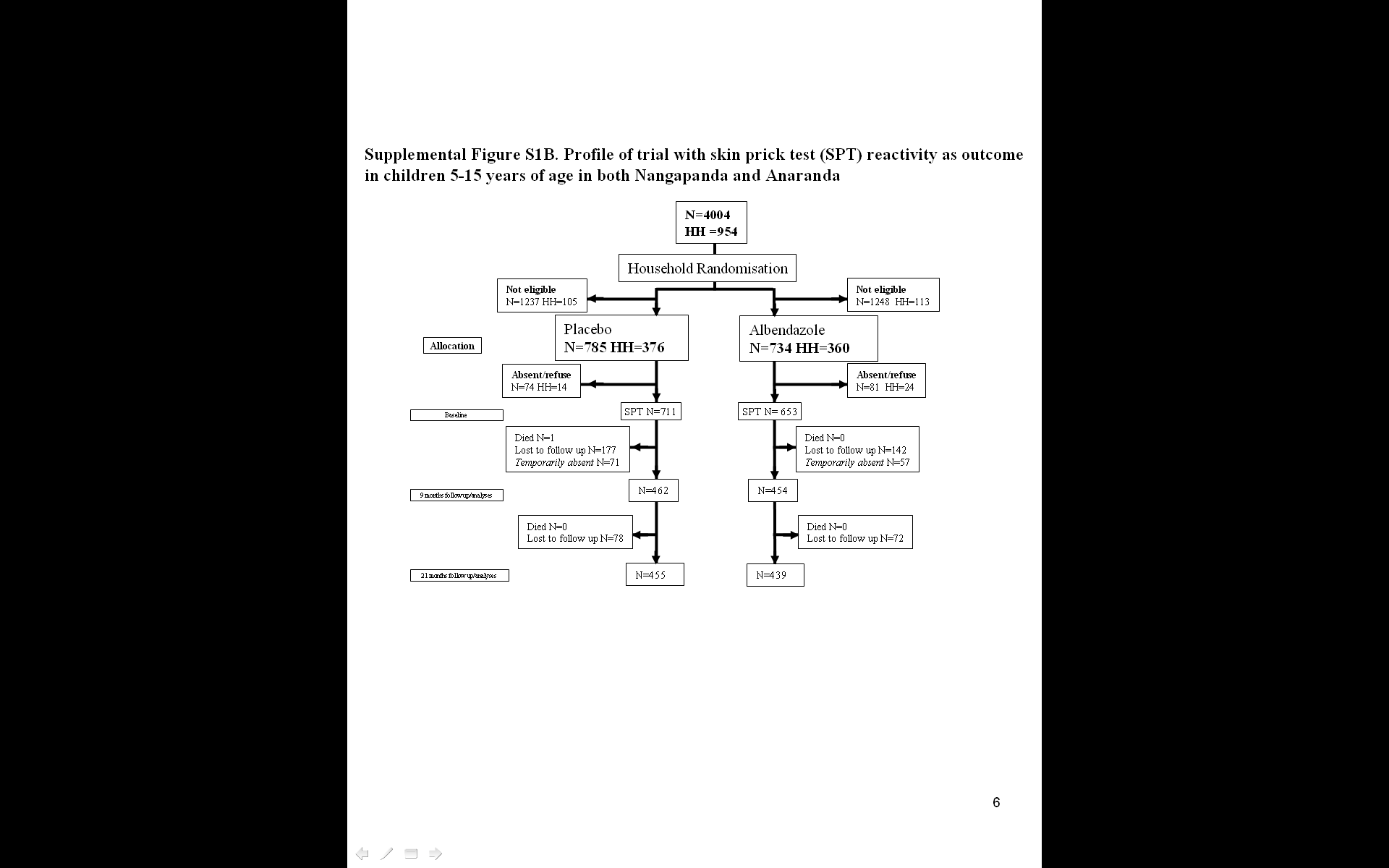


Figure S1C. Profile of trial with helminth infection as outcome in villages of Nangapanda and Anaranda


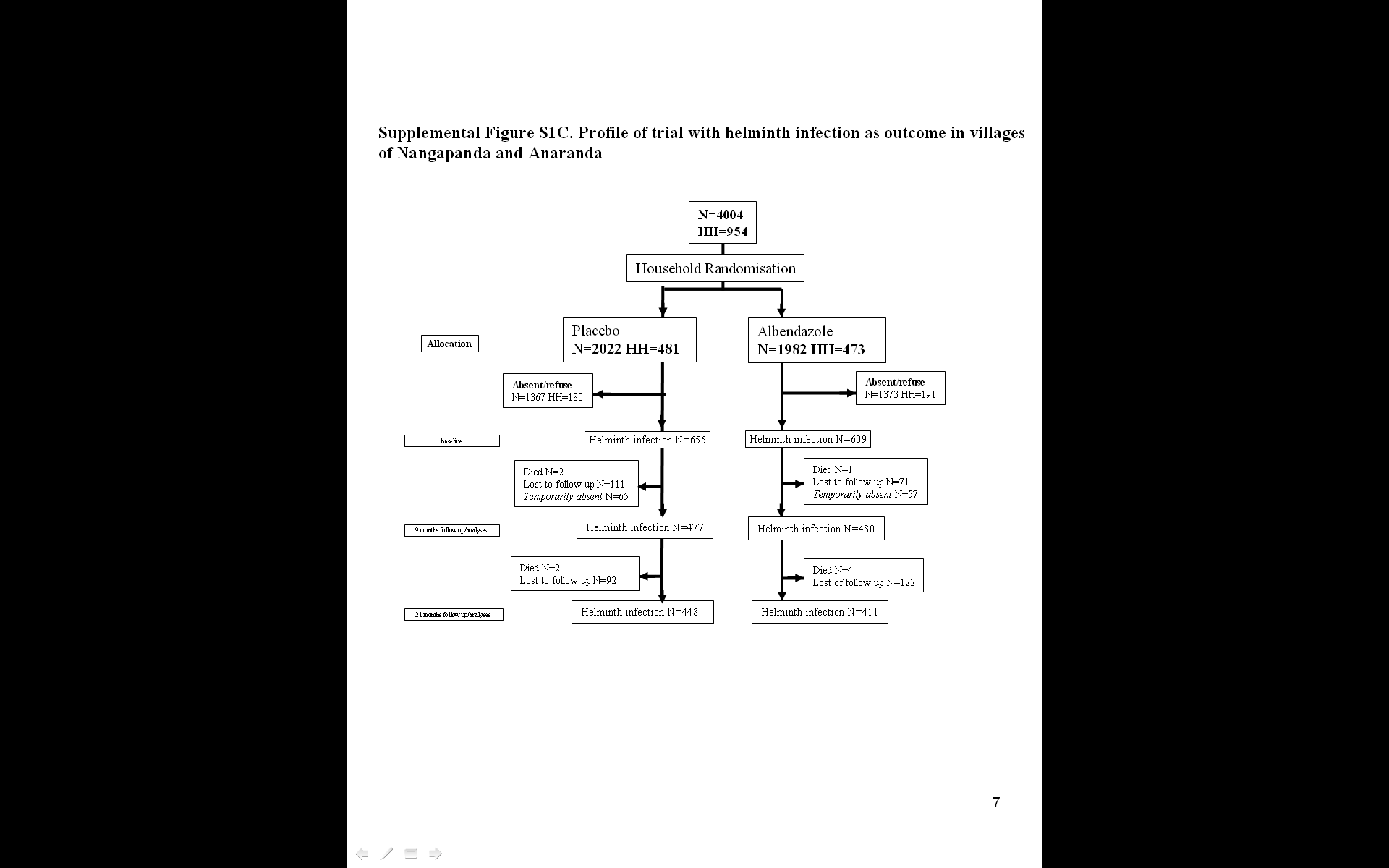

Supplement: Appendix S1 — Here we describe additional details on methods: study area and procedure, data collection on clinical symptoms, and detailed description of statistical models used. We provide tables on the effect of three monthly albendazole treatment on helminth infections, on reported fever and malaria-like symptoms, on reported clinical symptoms of allergy, and on body mass index. In addition, trial profiles of the separate outcomes are provided: malarial parasitemia, skin prick test, and helminth infection. (DOC) [file pone.0057899.s001.doc]
