# Supplementary material for: The Effect of Three-Monthly Albendazole Treatment on Malarial Parasitemia and Allergy: A Household-Based Cluster-Randomized, Double-Blind, Placebo-Controlled Trial
Source: PLoS One. 2013 Mar 19;8(3):e57899. doi: 10.1371/journal.pone.0057899 (PMC3602425; doi:10.1371/journal.pone.0057899)
Supplement: Checklist S1 — CONSORT Checklist. (DOC) [file pone.0057899.s002.doc]

**Table 1: CONSORT 2010 checklist of information to include when reporting a cluster randomised trial**

| **Section/Topic** | **Item No** | **Standard Checklist item** | **Extension for cluster designs** | **Section** |
| --- | --- | --- | --- | --- |
| **Title and abstract** | | | |  |
|  | 1a | Identification as a randomised trial in the title | Identification as a cluster randomised trial in the title | Title |
|  | 1b | Structured summary of trial design, methods, results, and conclusions (for specific guidance see CONSORT for abstracts)[[1]](#endnote-2),[[2]](#endnote-3) | See table 2 | Title and abstract |
| **Introduction** | | | |  |
| **Background and objectives** | 2a | Scientific background and explanation of rationale | Rationale for using a cluster design | Introduction |
|  | 2b | Specific objectives or hypotheses | Whether objectives pertain to the the cluster level, the individual participant level or both | Introduction |
| **Methods** | | | |  |
| **Trial design** | 3a | Description of trial design (such as parallel, factorial) including allocation ratio | Definition of cluster and description of how the design features apply to the clusters | Methods |
|  | 3b | Important changes to methods after trial commencement (such as eligibility criteria), with reasons |  | **-** |
| **Participants** | 4a | Eligibility criteria for participants | Eligibility criteria for clusters | Methods |
|  | 4b | Settings and locations where the data were collected |  | Methods and in supplementary appendix S1 |
| **Interventions** | 5 | The interventions for each group with sufficient details to allow replication, including how and when they were actually administered | Whether interventions pertain to the cluster level, the individual participant level or both | Methods and in supplementary appendix S1 |
| **Outcomes** | 6a | Completely defined pre-specified primary and secondary outcome measures, including how and when they were assessed | Whether outcome measures pertain to the cluster level, the individual participant level or both | Methods |
|  | 6b | Any changes to trial outcomes after the trial commenced, with reasons |  | **-** |
| **Sample size** | 7a | How sample size was determined | Method of calculation, number of clusters(s) (and whether equal or unequal cluster sizes are assumed), cluster size, a coefficient of intracluster correlation (ICC or *k*), and an indication of its uncertainty | Methods and findings |
|  | 7b | When applicable, explanation of any interim analyses and stopping guidelines |  | Protocol S1 and in supplementary appendix S1 |
| **Randomisation:** | | | |  |
| **Sequence generation** | 8a | Method used to generate the random allocation sequence |  | Methods |
|  | 8b | Type of randomisation; details of any restriction (such as blocking and block size) | Details of stratification or matching if used | Methods |
| **Allocation concealment mechanism** | 9 | Mechanism used to implement the random allocation sequence (such as sequentially numbered containers), describing any steps taken to conceal the sequence until interventions were assigned | Specification that allocation was based on clusters rather than individuals and whether allocation concealment (if any) was at the cluster level, the individual participant level or both | Methods and in supplementary appendix S1 |
| **Implementation** | 10 | Who generated the random allocation sequence, who enrolled participants, and who assigned participants to interventions | Replace by 10a, 10b and 10c | Methods and in supplementary appendix S1 |
|  | 10a |  | Who generated the random allocation sequence, who enrolled clusters, and who assigned clusters to interventions | Methods and in supplementary appendix S1 |
|  | 10b |  | Mechanism by which individual participants were included in clusters for the purposes of the trial (such as complete enumeration, random sampling) | Methods |
|  | 10c |  | From whom consent was sought (representatives of the cluster, or individual cluster members, or both), and whether consent was sought before or after randomisation | Methods |
|  |  |  |  |  |
| **Blinding** | 11a | If done, who was blinded after assignment to interventions (for example, participants, care providers, those assessing outcomes) and how |  | Methods and in supplementary appendix S1 |
|  | 11b | If relevant, description of the similarity of interventions |  | - |
| **Statistical methods** | 12a | Statistical methods used to compare groups for primary and secondary outcomes | How clustering was taken into account | Methods and in supplementary appendix S1 |
|  | 12b | Methods for additional analyses, such as subgroup analyses and adjusted analyses |  | Methods and in supplementary appendix S1 |
| **Results** | | | |  |
| **Participant flow (a diagram is strongly recommended)** | 13a | For each group, the numbers of participants who were randomly assigned, received intended treatment, and were analysed for the primary outcome | For each group, the numbers of clusters that were randomly assigned, received intended treatment, and were analysed for the primary outcome | Results and in supplementary appendix S1 |
|  | 13b | For each group, losses and exclusions after randomisation, together with reasons | For each group, losses and exclusions for both clusters and individual cluster members | Results and in supplementary appendix S1 |
| **Recruitment** | 14a | Dates defining the periods of recruitment and follow-up |  | Methods |
|  | 14b | Why the trial ended or was stopped |  | Methods |
| **Baseline data** | 15 | A table showing baseline demographic and clinical characteristics for each group | Baseline characteristics for the individual and cluster levels as applicable for each group | Table 1 |
| **Numbers analysed** | 16 | For each group, number of participants (denominator) included in each analysis and whether the analysis was by original assigned groups | For each group, number of clusters included in each analysis | Results |
| **Outcomes and estimation** | 17a | For each primary and secondary outcome, results for each group, and the estimated effect size and its precision (such as 95% confidence interval) | Results at the individual or cluster level as applicable and a coefficient of intracluster correlation (ICC or k) for each primary outcome | Results |
|  | 17b | For binary outcomes, presentation of both absolute and relative effect sizes is recommended |  | Results |
| **Ancillary analyses** | 18 | Results of any other analyses performed, including subgroup analyses and adjusted analyses, distinguishing pre-specified from exploratory |  | Results and in supplementary appendix S1 |
| **Harms** | 19 | All important harms or unintended effects in each group (for specific guidance see CONSORT for harms[[3]](#endnote-4)) |  | Results and in supplementary appendix S1 |
| **Discussion** | | | |  |
| **Limitations** | 20 | Trial limitations, addressing sources of potential bias, imprecision, and, if relevant, multiplicity of analyses |  | Discussion |
| **Generalisability** | 21 | Generalisability (external validity, applicability) of the trial findings | Generalisability to clusters and/or individual participants (as relevant) | Discussion |
| **Interpretation** | 22 | Interpretation consistent with results, balancing benefits and harms, and considering other relevant evidence |  | Discussion |
| **Other information** | | |  |  |
| **Registration** | 23 | Registration number and name of trial registry |  | Abstract and methods |
| **Protocol** | 24 | Where the full trial protocol can be accessed, if available |  | Protocol S1 file |
| **Funding** | 25 | Sources of funding and other support (such as supply of drugs), role of funders |  | Abstract |

**Table 2: Extension of CONSORT for abstractsError: Reference source not found^,Error: Reference source not found^ to reports of cluster randomised trials**

| **Item** | **Standard Checklist item** | **Extension for cluster trials** |
| --- | --- | --- |
| **Title** | Identification of study as randomised | **Identification of study as cluster randomised (yes, p1 line 1-3)** |
| **Trial design** | Description of the trial design (e.g. parallel, cluster, non-inferiority) | **p3, line 49.** |
| **Methods** |  |  |
| **Participants** | Eligibility criteria for participants and the settings where the data were collected | **Eligibility criteria for clusters (yes, p3 line 50-52)** |
| **Interventions** | Interventions intended for each group | **p3, line 51-52** |
| **Objective** | Specific objective or hypothesis | **Whether objective or hypothesis pertains to the cluster level, the individual participant level or both (yes, p3 line 47-48)** |
| **Outcome** | Clearly defined primary outcome for this report | **Whether the primary outcome pertains to the cluster level, the individual participant level or both (yes, p3 line 60-63)** |
| **Randomization** | How participants were allocated to interventions | **How clusters were allocated to interventions (yes, p3 line 50-51)** |
| **Blinding (masking)** | Whether or not participants, care givers, and those assessing the outcomes were blinded to group assignment | **p3, line 52-53** |
| **Results** |  |  |
| **Numbers randomized** | Number of participants randomized to each group | **Number of clusters randomized to each group (yes, p3 line 51-52)** |
| **Recruitment** | Trial status[[4]](#footnote-2) |  |
| **Numbers analysed** | Number of participants analysed in each group | **Number of clusters analysed in each group (yes, p3 line 51-52,58)** |
| **Outcome** | For the primary outcome, a result for each group and the estimated effect size and its precision | **Results at the cluster or individual participant level as applicable for each primary outcome (yes, p3 line 59-64)** |
| **Harms** | Important adverse events or side effects | **p3, line 64** |
| **Conclusions** | General interpretation of the results | **p3 line 65-67** |
| **Trial registration** | Registration number and name of trial register | **p1 line 4, p3 line 68-69)** |
| **Funding** | Source of funding | **p3 line 68** |
|  |  |  |

**REFERENCES**

1. Hopewell S, Clarke M, Moher D, Wager E, Middleton P, Altman DG, et al. CONSORT for reporting randomised trials in journal and conference abstracts. *Lancet* 2008, 371:281-283 [↑](#endnote-ref-2)
2. Hopewell S, Clarke M, Moher D, Wager E, Middleton P, Altman DG at al (2008) CONSORT for reporting randomized controlled trials in journal and conference abstracts: explanation and elaboration. *PLoS Med* 5(1): e20 [↑](#endnote-ref-3)
3. Ioannidis JP, Evans SJ, Gotzsche PC, O'Neill RT, Altman DG, Schulz K, Moher D. Better reporting of harms in randomized trials: an extension of the CONSORT statement. *Ann Intern Med* 2004; 141(10):781-788. [↑](#endnote-ref-4)
4. Relevant to Conference Abstracts [↑](#footnote-ref-2)
