# Supplementary material for: The Effect of Three-Monthly Albendazole Treatment on Malarial Parasitemia and Allergy: A Household-Based Cluster-Randomized, Double-Blind, Placebo-Controlled Trial
Source: PLoS One. 2013 Mar 19;8(3):e57899. doi: 10.1371/journal.pone.0057899 (PMC3602425; doi:10.1371/journal.pone.0057899)
Supplement: Protocol S1 — Trial Protocol. (DOC) [file pone.0057899.s003.doc]

# Study program:

# ImmunoSPIN program in Indonesia “Parasitic Infections and Inflammatory Diseases: The web of immune responses, host genetics and environmental exposure”

# *Trial protocol adjusted for manuscript:*

# *The effect of three-monthly albendazole treatment on malarial parasitemia and allergy: A household-based cluster-randomized, double-blind, placebo-controlled trial*

**Principal investigator in Indonesia:**

Dr. Taniawati Supali (PhD)

Department of Parasitology

Faculty of Medicine, University of Indonesia

Salemba Raya 6

Jakarta 10430/ Indonesia

Tel: +62 (21) 3914607

Fax +62 (21) 39832018

E-mail: [taniawati@yahoo.](mailto:hoerauf@parasit.meb.uni-bonn.de)com

**Principal investigator in the Netherlands:**

Professor Maria Yazdanbakhsh (PhD)

Department of Parasitology

Leiden University Medical Center

2333 ZA Leiden

Tel.: +31 (71) 5265072

FAX: +31 (71) 5266907

E-mail: m.yazdanbakhsh@lumc.nl

**Field investigators:**

Dr. Aprilianto Eddy Wiria (MD)

Department of Parasitology

Faculty of Medicine, University of Indonesia

Salemba Raya 6

Jakarta 10430/ Indonesia

Tel: +62 (21) 3914607

Fax +62 (21) 39832018

Dr. Firdaus Hamid (MD)

Department of Microbiology

Medical Faculty, Hasanuddin University

Jl. Perintis Kemerdekaan

Kampus Tamalanrea KM10

Makassar 90245, Indonesia

Dr. Linda J Wammes (M.Sc, MD)

Department of Parasitology

Leiden University Medical Center

2333 ZA Leiden

E-mail: l.j.wammes@lumc.nl

**Research team:**

Dr. Adrian Luty

Radboud University Nijmegen Medical Centre

Department of Medical Microbiology

Medical Parasitology-268, MMB-NCMLS, PO Box 9101, 6500 HB Nijmegen

Tel.: +3124 3613663

Fax: +3124 3614666

Dr. Erliyani Sartono

Department of Parasitology

Leiden University Medical Center

2333 ZA Leiden

Tel.: +31(71) 5265072

FAX: +31 (71) 5266907

Dr. Bertrand Lell

Laboratoire de Recherches
Hôpital Albert Schweitzer
B.P.118
Lambaréné, Gabon

Tel: +241 07989191

Prof. Inge Sutanto

Department of Parasitology

Faculty of Medicine, University of Indonesia

Salemba Raya 6

Jakarta 10430/ Indonesia

Tel: +62 (21) 3914607

Fax +62 (21) 39832018

Dr. Sitti Wahyuni

Department of Parasitology

Medical Faculty, Hassanuddin University

Jl. Perintis Kemerdekaan, kampus Tamalanrea KM 10

Makassar-90245, Indonesia

Tel.: + 62 411 586010

Fax: + 62411 586297

Dr. Iwan Ariawan

Department of Biostatistics,

School of Public Health,

University of Indonesia,

Jakarta, Indonesia

Tel: +62 (21) 7863473

Fax: + 62 (21) 7871636

Dr. Gary Brice

LCDR MSC USNR, United States Navy Medical Research Unit #2, Jakarta

FPO AP 96520-8132

Tel.:+ 62-21-421-4457 ext 1121

Fax:+ 62-21-424-4507

**Responsible clinician for adverse events reporting:**

Dr. Bertrand Lell

**Publication committee:**

Professor Maria Yazdanbakhsh

Dr. Taniawati Supali

Dr. Erliyani Sartono

Dr. Adrian Luty

**Background:**

Major intervention programs have been launched, which use mass drug administration (MDA), often given annually and over protracted periods, to control STH. However, the evidence base is lacking on how effective MDA can be and whether it might result in the emergence of pathological conditions, as helminth infections are thought to have profound effects on the immune system.

Parasitic helminths have strong immunomodulatory activities affecting responses to third party antigens. In many parts of the developing world, malarial and helminth infections are co-endemic and many children are infected by both parasites. Investigation on the immunological associations between helminth infections and malarial parasites in co-endemic areas hold the key to answer the question whether helminths increase suppressory immune responses and increase susceptibility to malarial parasites on the one hand but protect from cerebral malaria on the other. Indonesia is a country in transition from one with high burden of infection and traditional lifestyle to one where infectious diseases will be controlled and modern lifestyle will take over bringing along an increase in inflammatory diseases such as allergies. So far most studies analyzing the effect of helminths on immune responses, malaria or allergies have been cross sectional. There are only two recent longitudinal study of the effect of anti helminths on allergy with a randomised placebo controlled design and none studying malaria outcomes; such studies are needed to establish the role that helminths play in suppressing immune responses and affecting malaria or allergies. For co-infections with malarial parasites, detrimental or protective effects of helminth infections will change policies regarding helminth control programs as would information on the mechanistic pathways such as immune responses that can provide valuable information for vaccines and drug design that aim to control infection or disease. Equally important is the results generated from studies involving the effect of helminths on inflammatory diseases such as allergies. These are diseases of populations with Western life style and as Indonesia is moving rapidly towards stronger economy and consequent increased urbanisation and adoption of Western life style, this program is getting prepared for what are expected to form serious future public health problems. The global initiative to control helminth infections worldwide is based on assumptions that these infections affect child health. Growth and cognition are thought to be adversely affected. However, a recent review challenges the consistency of such findings.

Altogether, well-controlled randomised trials are needed to provide us with robust data regarding the effects that helminth infections have on health and disease.

**Key references**

Bethony J, Brooker S, Albonico M et al. Soil-transmitted helminth infections: ascariasis, trichuriasis, and hookworm. Lancet 2006; 367: 1521-1532.

Maizels RM, Yazdanbakhsh M. Immune regulation by helminth parasites: cellular and molecular mechanisms. Nat Rev Immunol 2003; 3:733-44.

Nacher M, Singhasivanon P, Silachamroon U et al. Helminth infections are associated with protection from malaria-related acute renal failure and jaundice in Thailand. Am J Trop Med Hyg 2001; 65:834-6.

van Den Biggelaar AH, van Ree R, Rodrigues LC et al. Decreased atopy in children infected with Schistosoma haematobium: a role for parasite-induced interleukin-10. Lancet 2000; 356:1723-7.

Cooper PJ, Chino ME, Rodrigues LC, Ordonez M, Strachan D, Griffin GE, Nutman TB. Reduced risk of atopy among school age children infected with geohelminth parasites in a rural area of tropics. J Allergy Clin Immunol 2003;111:995-1000.

Cooper PJ, Chico ME, Vaca MG, Moncayo AL, Bland JM, Mafla E, Sanchez F, Rodrigues LC, Strachan DP, Griffin GE. [Effect of albendazole treatments on the prevalence of atopy in children living in communities endemic for geohelminth parasites: a cluster-randomised trial.](http://www.ncbi.nlm.nih.gov/pubmed/16698413) Lancet. 2006;367:1598-603.

Murray MJ, Murray AB, Murray MB, Murray CJ. Parotid enlargement, forehead edema, and suppression of malaria as nutritional consequences of ascariasis. Am J Clin Nutrit 1977;**30**:2117–2121

**Trial objectives and purpose:**

- To assess the effect of albendazole on prevalence of malarial parasitemia and on fever and malarial-like symptoms.
- To assess the effect of albendazole on prevalence of SPT reactivity to allergens and symptoms of allergy.
- To assess the effect of albendazole on prevalence of soil-transmitted helminths.

**Trial design**

Household-based cluster-randomized, double-blind, placebo-controlled trial. Household clustering will be used in order to maximally interrupt the transmission of helminths.

**Trial Endpoints**:

***Primary outcomes***

- prevalence of malarial parasitemia
- prevalence skin prick test (SPT) reactivity to allergens
- prevalence of soil-transmitted helminths

***Additional outcomes***

- fever and additional malaria-like symptoms
- symptoms of allergy
- body mass index

**Trial treatment:**

Albendazole (400 mg) and identical pill as placebo were manufactured by PT Indofarma Pharmaceutical, Bandung, Indonesia. The treatment will be provided every three months for a period of 21 months after the start of treatment.

**Randomization and blinding**

The houses will be marked by Global Positioning System (GPS) system to allow maps to be generated using ARC GIS 9 software (ESRI, USA). Individual’s geographical coordinates will be assigned as well as centre coordinates for households. Computer-aided block randomization by household, using Random Allocation Software will be used to assign treatment groups. The treatment will be coded as A or B and the code will be concealed from investigators and patients. Labels with the study subject ID will be printed from a computer database and attached to the appropriate strip of treatment by the study team located in Jakarta without the involvement of the field study investigators.

A number of nested studies will be conducted which will also use the Random Allocation software to apply to the treatment A and B arms and generate within each of these arms, sub samples for cellular immunological study. The treatment code will be de-blinded when all data needed for analyses are entered into the database.

**Selection of study subjects**

***Inclusion criteria***

Subjects of both sexes, aged 2 years and older, in good health, without any clinical condition requiring chronic medication and who have given informed consent (written or thumb print) will be recruited into the trial.

***Exclusion criteria***

Children younger than 2 years of age

Pregnant women

Subjects with clinical condition requiring chronic medication

No informed consent

### Procedures for collection of samples

**Methods and timing of assessments**

***Malaria parasite assessment by microscopy***- at pre treatment and at 3, 6, 9, 12, 15, 18 and 21 months after the start of treatment.

Thick and thin blood smears will be prepared from finger prick blood. The slides will be stained with Giemsa and examined microscopically for all species of malaria parasites according to WHO SOP. The technicians reading the malaria slides will be certified at the US-Naval Medical Research Unit #2 (US-NAMRU2) in Jakarta. The outcome will be positive or negative to assess prevalence.

***Malaria parasite assessment by Polymerase Chain Reaction (PCR)-*** at pre treatment and at 9 and 21 months after the start of treatment.

Blood kept frozen (at -20^o^ C) will be used for DNA extraction and analysis by PCR according to published protocols (Wiria et al., BMC Infect Dis. 2010 Mar 25;10:77 and Hamid et al., BMC Infect Dis. 2011 Apr 1;11:83). The test will be undertaken at LUMC.

***Helminth parasite assessment by microscopy***- at pre treatment and at 9 and 21 months after the start of treatment.

Stool containers will be distributed and collected by health workers and delivered to the field laboratory. Technicians trained and certified in Jakarta will perform the microscopic examination of the stool samples (Wiria et al., BMC Infect Dis. 2010 Mar 25;10:77 and Hamid et al., BMC Infect Dis. 2011 Apr 1;11:83).

***Helminth parasite assessment by PCR***- at pre treatment and at 9 and 21 months after the start of treatment.

Fresh stool samples will be kept frozen (at -20^o^ C) and used for DNA extraction and analysis by PCR to detect *Ascaris lumbricoides, Necator americanus and Ancylostoma duodenale* as well as *Strongyloides stercoralis*, according to published protocols (Wiria et al., BMC Infect Dis. 2010 Mar 25;10:77 and Hamid et al., BMC Infect Dis. 2011 Apr 1;11:83). The test will be undertaken at LUMC.

***Fever and additional malaria-like symptom registration*** by questionnaires designed based on WHO recommended criteria at monthly visits by trained health workers. Fever will be measured by assessing oral temperature using digital thermometers.

The forms to record fever and additional malaria-like symptoms are appended (see appendix 1), will be used by trained health workers at monthly visits to households. Each health worker will be responsible for 20 houses. History of fever, malaria like-symptoms, health care visits and medications used are recorded within one month period. Individuals with fever and/or additional malaria-like symptoms within two days and at the time of visit are referred to the local primary health center (Puskesmas) for further assessment.

***Skin prick testing***- at baseline pre treatment and at 9 and 21 months post treatment assessed by one investigator. The skin prick testing (SPT) to allergens *Dermatophagoides pteronyssinus* and *farinae*, *Blatella germanica* is performed according to protocol published (Hamid et al., BMC Infect Dis. 2011 Apr 1;11:83). The study of allergic outcomes aims to look at school-age children and therefore this test will be restricted to school-age children in our study households.

***ISAAC questionnaire administration***- at baseline pre treatment and at 21 months after the start of treatment by one investigator

The ISAAC questionnaires developed specifically for study area during EU funded project GLOFAL ([www.glofal.org](http://www.glofal.org/)) will be used along with visual material to assess the prevalence of reported allergic symptoms (Asthma, Rhinitis and Eczema).

The study of allergic outcomes aims to look at school-age children and therefore this test will be restricted to school-age children in our study households.

***Anthropometry assessment***-at baseline pre treatment and at 9 and 21 months after the start of treatment.

Height and weight will be measured using scales (standard SECA tools).

***Blood sampling-*** at baseline pre treatment and at 9 and 21 months post treatment, 6 mls of peripheral blood collected into heparin tubes (BD vaccutainers) by experienced medical staff for use in immunological work. Plasma samples will be separated for measurement of antibody levels

**Assessment of adverse events**

Although albendazole is a well established safe drug, the study population will be informed to report any adverse reactions to field physicians and health workers. Adverse events spontaneously reported by the patient or observed by the investigators, will be monitored throughout the study. The reports will be sent to safety clinician responsible for adverse events reporting and assessed whether it is a trial related adverse event.

**Data management**

The database will be designed in MS Access and managed at LUMC but the latest database will be sent to the PI in Indonesia at weekly intervals as read only file. All data collected in the field at the monthly follow ups of fever and additional malaria-like symptoms will be entered by two trained data entry personnel after the forms are inspected and approved by study investigator. For each data series, procedure for double entry, quality control and coherence queries procedures will be implemented systematically in order to detect errors and/or missing data. After integration of all the data corrections on the whole data base, the data base will be locked and saved before it is transferred to the trial statistician. Each step of the process will be controlled by implementation of individual passwords and/or regular back-ups, in order to allow access to the data base and to ensure its integrity.

**Confidentiality of individual data**

Participant confidentiality will be strictly safeguarded by the participating investigators and their staff. This confidentiality is extended to cover testing of biological samples and possible future genetic tests in addition to the clinical information relating to participants. The study protocol, documentation, data, and all other information generated will be held in strict confidence. No information concerning the study or the data will be released to any unauthorized third party.

**Statistics**

***Sample size calculation-*** The sample size calculation document was prepared by statisticians from LUMC and is provided in a document in appendix 2.

#### Analyses of Trial data- The LUMC will conduct the analyses of trial data for manuscripts approved by the publication committee. At a minimum, the trial manuscripts are expected to address the primary and secondary outcomes as well as the additional outcomes listed in the protocol.

***Statistical methods***- The appropriate statistical methods for longitudinal data and repeated measurements will be used. As the data are very complex, appropriate modeling will be needed in close collaboration with biostatisticians at LUMC to analyze the data.

**Laboratory methods**

The laboratory methods used have been published in 2 protocol papers. (Wiria et al., BMC Infect Dis. 2010 Mar 25;10:77 and Hamid et al., BMC Infect Dis. 2011 Apr 1;11:83).

**Quality control**

Several measures will be taken to assure a high level of quality control in all aspects of the study.

Questionnaires: health workers will attend regular training sessions to be trained and re trained by study investigators in order to keep to standard administration of questionnaires that record malaria-like symptoms.

All pipettes will be calibrated twice a year at LUMC and returned to the field laboratory.

The technicians conducting malaria and helminth assessment will be trained and re trained in Jakarta and in the field twice a year.

Double entry of selected fields will be undertaken, to assure that errors in data entry are minimized and the final dataset is as clean as possible.

**Supplies**

All supplies needed for the study not available in Jakarta will be shipped from the Netherlands to Jakarta and from Jakarta to Ende. The supplies will be the same (ie same company and product number) throughout the study.

The checklist of reagents will be drawn every 3 months and orders will be placed by fax or email (if internet connection works properly).

Storage room will be checked and lists will be updated at one monthly interval with study investigators being responsible for the updates.

**Sample labeling**

The labels will be of a quality that remains intact under all temperatures needed in the study. Before taking any sample, correct tubes and accessories shall be checked, and the tubes labeled accordingly. A label will contain ID number and a letter from alphabet to indicate the pre and post treatment follow up time points.

**Sample storage**

-Plasma samples will be stored at -20 ^o^C

-Stool samples will be stored at room temperature (formalin preservation), -20 ^o^C (fresh stool for

PCR)

-Freezer temperature will be recorded regularly with thermometer.

**Sample transport**

Plasma, and stools will be transported to Jakarta on dry ice that will be flown into Flores via Bali or Jakarta.

**Time frame**

The study has a level of funding that was cut down considerably by the grant giving body. The aim was originally, when grant was written to conduct the study for a period of 3 years. However, financial constrains could lead to the earlier termination of the study. In addition, if the results from one and two year interim analysis indicate that it is not ethical to withhold albendazole to half of the study population, the trial will be ended.

**Ethical issues and approval**

The study will be submitted to the Committee of the Medical Research Ethics of the Faculty of Medicine of the University of Indonesia and to the LUMC Committee of Medical Ethics. Further, efforts will be made to ensure approval of the local village leaders and parents/guardians of the study children. To ensure ethical conduct of the trial, all the clinical investigators will follow courses on trial methods (ErasmusMC, Netherlands), courses on GCP and GLP organized by EU funded projects GLOFAL and COINFECT.

**Informed Consent Process**

The principles of informed consent in the current edition of the Declaration of Helsinki will be implemented before any protocol-specified procedures or interventions are carried out. Information will be given in both oral and written form whenever possible. Independent witnesses will be required to attest that illiterate parents/guardians of potential participants have understood the contents of the informed consent.

It is expected that there will be extensive contact between the study team and the populations involved that should lead to the development of mutual trust and the establishment of an ongoing informed consent process attempting to address issues related to interventional studies in resource-limited settings. Many discussions with local community leaders, heads of families and citizens through group meetings, and more limited group interviews will be undertaken prior to trial start.

All relevant information on the possible risks such as discomfort and pain during blood sampling, the acceptance of health workers at monthly intervals they need to honor etc, will be explained. In all cases, the investigator will give the participants ample opportunity to inquire about the details of the trial and to ask any questions before dating and signing the consent forms. Informed consent approved by the ethics committee, will be signed or thumb-printed and dated by the participant’s parent/guardian and by the person who conducted the informed consent discussion. Thumb printing will be used for illiterate persons only

**Appendix 1**

**The forms to record fever and additional malaria-like symptoms**

Date of Visit: ___ ___ . ___ ___ . ___ ___ ___ ___

Name of Subject: _________________________________ Subject ID: ___ ___ ___ ___

Village: ___ Household: ___ ___ ___ Name of Cadre: _______________

1. **Was subject ill since last visit ?** ⁮ Yes ⁮ No

1a. **Symptoms:**

⁮ Fever (including Chills and Sweating)

⁮ Headache

⁮ Fatigue (including Myalgia and Arthralgia)

⁮ Vomiting

⁮ other (specify): _________________

1b. **How long ago ?** _____ days ago / ⁮ currently ill?

1c. **Duration of symptoms** : _____ days

1d. **Temperature (if fever or history of fever):** _________ °C

2. **Did subject take treatment for illness ?** ⁮ Yes ⁮ No

2a. **Who gave treatment ?** ⁮ self-medication ⁮ puskesmas ⁮ doctor ⁮ other

2b. **Name of drug (incl. traditional herbs)**: ___________________________________________

3. **Did subject visit health care center since last visit ?**  ⁮ Yes ⁮ No

3a. **Which ?** ⁮ puskesmas ⁮ doctor ⁮ hospital ⁮ other

3b. **Date of visit:** ___ ___ . ___ ___ . ___ ___ ___ ___

3c. **hospitalized?** ⁮ Yes ⁮ No

4. **Notes:**

|  |
| --- |

**Appendix 2**

**Sample Size Calculation for ImmunoSPIN project**

Calculation are based on data from pilot study and from literature.

*For all calculations power is set at 0.90 and significance level at 0.05*

SKIN PRICK TEST

The 4^th^ column gives the number of people needed in either treatment arm to find a post treatment SPT prevalence as predicted in column 2. In column 5 the number of individuals in either treatment arm needed, presuming that 20% of the people may be lost to follow up (LTFU). The last column gives the total number of enrolments before randomization under this condition.

| Prevalence (P_0_)  -control group- | Predicted prevalence (P_1_)  - treatment group- | RR | N = treated | If 20% LTFU | Total enrolments (before randomization) |
| --- | --- | --- | --- | --- | --- |
| **HDM 15%** | 17.5% | 1.2 | 4567 | 5720 | 11440 |
|  | 20% | 1.3 | 1212 | 1515 | 3030 |
|  | **22.5%** | **1.5** | **567** | **709** | **1418** |
|  | **25%** | **1.7** | **334** | **418** | **835** |
|  | 27.5% | 1.8 | 223 | 279 | 558 |
|  | 30% | 2 | 161 | 242 | 484 |
|  |  |  |  |  |  |
| **Cockr. 35%** | 37.5% | 1.07 | 7771 | 9714 | 19428 |
|  | 40% | 1.14 | 1969 | 2461 | 4922 |
|  | 42.5% | 1.21 | 885 | 1106 | 2212 |
|  | **45%** | **1.29** | **502** | **628** | **1256** |
|  | **47.5%** | **1.36** | **324** | **405** | **810** |
|  | 50% | 1.43 | 226 | 283 | 566 |

MALARIAL PARASITEMIA

| Prevalence (P_0_)  -control group- | Predicted prevalence (P_1_)  - treatment group- | RR | N = treated | If 20% LTFU,  Treatm. failure | Total enrolments (before randomization) |
| --- | --- | --- | --- | --- | --- |
| **20%** | 17.5% | 0.88 | 5122 | 6403 | 12805 |
|  | **15%** | **0.75** | **1212** | **1515** | **3030** |
|  | **14%** | **0.70** | **822** | **1028** | **2056** |
|  | 12.5% | 0.63 | 507 | 634 | 1268 |
|  | 10% | 0.5 | 266 | 333 | 666 |
|  |  |  |  |  |  |
| **10%** | 7.5% | 0.75 | 2684 | 3355 | 6710 |
|  | 6.75% | 0.67 | 1525 | 1906 | 3812 |
|  | **6%** | **0.60** | **965** | **1206** | **2412** |
|  | 12.5% | 1.25 | 3356 | 4206 | 8412 |
|  | 13.75% | 1.38 | 1562 | 1953 | 3906 |
|  | **15%** | **1.5** | **918** | **1148** | **2295** |

**Key reference**

van den Biggelaar AH, Borrmann S, Kremsner P, Yazdanbakhsh M. [Immune responses induced by repeated treatment do not result in protective immunity to Schistosoma haematobium: interleukin (IL)-5 and IL-10 responses.](http://www.ncbi.nlm.nih.gov/pubmed/12404164) J Infect Dis. 2002;186:1474-82.

van den Biggelaar AH, Rodrigues LC, van RR, van der Zee JS, Hoeksma-Kruize YC, Souverijn JH et al. Long-term treatment of intestinal helminths increases mite skin-test reactivity in Gabonese schoolchildren. J Infect Dis 2004;189:892-900.
